# Pd@[*n*Bu_4_][Br] as a Simple Catalytic System for *N*-Alkylation Reactions with Alcohols

**DOI:** 10.3390/molecules21081042

**Published:** 2016-08-10

**Authors:** Bastien Cacciuttolo, Oana Pascu, Cyril Aymonier, Mathieu Pucheault

**Affiliations:** 1 CNRS, Université de Bordeaux, ISM, UMR 5255, ISM, 351 cours de la libération, 33405 Talence Cedex, France; mathieu.pucheault@u-bordeaux.fr; 2 CNRS, Université de Bordeaux, ICMCB, UPR 9048, ICMCB-CNRS, 87 avenue du Dr Albert Schweitzer, 33608 Pessac Cedex, France; aymonier@icmcb-bordeaux.cnrs.fr

**Keywords:** *N-*alkylation, nanoparticle, onium salt

## Abstract

Palladium nanoparticles, simply and briefly generated in commercial and cheap onium salts using supercritical carbon dioxide, have been found to be an effective catalytic system for additive free *N-*alkylation reaction using alcohols via cascade oxidation/condensation/reduction steps.

## 1. Introduction

**Scheme 1:**
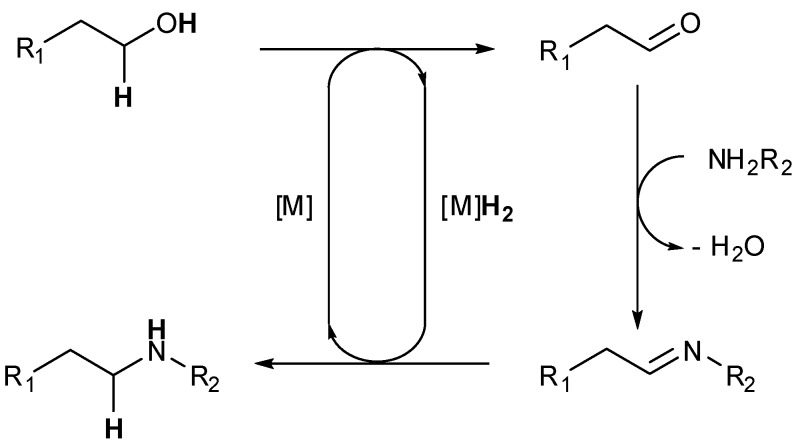
Amination of alcohols by borrowing-hydrogen method, cascade oxidation/imine formation/reduction.

The amine function plays a major role in many industrial fields such as materials, pharmaceuticals and agrochemicals [1,2,3]. This function is present not only in bioactive structures, such as amino acids and alkaloids, but also in numerous intermediates for polymers and dyes synthesis. *N-*alkylated amines are mainly obtained either by reaction between an amine and an alkyl halide [4] in the presence of stoichiometric amount of a base or by reductive amination [5] of ketone or aldehyde starting materials. Alternatively, products can be obtained from alcohols, avoiding the presence of potentially mutagenic alkylating reagents or carbonyl compounds as unstable starting materials. Several methods using direct nucleophilic substitution on the hydroxyl group can be considered. However, the main developed methodology relies on a “self-hydrogen transfer” reaction, the so called “hydrogen-borrowing” method (Scheme 1).

In a one pot synthesis, three distinct steps are carried out: first the oxidation step of the starting alcohol takes place, followed by formation of an imine which will finally be reduced to the *N-*alkyl amine, the hydrogen transfer being performed by the catalyst during oxidation and reduction steps. Water is the only by-product generated and the reaction could theoretically occur in the absence of hydrogen, thereby enhancing the system safety.

By using noble (Pd [6], Ru [7,8,9,10,11,12], Ir [13,14,15,16], Rh [17,18]) and non-noble metals (Cu [19,20,21], Fe [22,23]) as homogenous catalysts for the *N-*alkylation reaction, several efficient methods have been developed. The improvement of the reaction system allowing easy recycling of the catalyst has led to the emergence of numerous studies advocating the use of heterogeneous catalysts (Pd [24,25,26,27,28], Ru [29,30,31,32], Pt [33,34], Au [35,36,37,38,39,40], Ag [41,42], Ni [43,44,45], Mn [46], Cu [47,48,49], Fe [50,51]) with more or less efficiency.

The synthesis of nanoscaled materials has allowed for the evolution of a new field of catalysis. Indeed, at nanosize, the physicochemical properties of materials can change dramatically, due to a high specific surface area but also to a higher number of metal low valence favouring radical formations. It is known that noble metal nanoparticles are effective in many catalytic processes for both reduction and oxidation reactions and the trend is the preparation of hybrid noble metal nanocatalysts, as simple as can be. Ionic liquids (ILs), as solvents, stabilizers and reducing agents [52,53] are very attractive for an easy and clean synthesis of noble metal-based nanocatalysts [54]. Bringing together the properties of ILs (low surface tension, the presence of charges able to create an electrostatic shell around the metal core, a myriad of structures and so on) [55] with the advantages of supercritical fluids technology (homogeneous single phase reaction media, properties of both liquids—density and solvation capabilities—and gases—high diffusivity, low viscosity and zero surface tension, increased overall reaction rate) highly crystalline metal NPs embedded in a solid matrix and additionally free from any undesired organic parts can be prepared [56]. Among supercritical fluids, supercritical carbon dioxide (*sc*CO_2_) is especially attractive due to its low critical coordinates of 31 °C and 7.38 MPa and its accessibility, being abundant, cheap, inoffensive, and environmentally friendly, with also no liquid waste generation [57,58].

The present study is focused on the use of palladium NPs as hybrid nanocatalysts for the *N-*alkylation reaction. The Pd NPs are generated in onium salts (OSs) with the assistance of *sc*CO_2_ in the absence of molecular solvent. The resulting easy-to-handle powdered OS embedded Pd NPs are stable towards air and moisture and display good catalytic activities in the green synthesis of *N-*alkylated anilines.

## 2. Results

### 2.1. Synthesis and Characterization of the Catalyst

**Figure 1:**
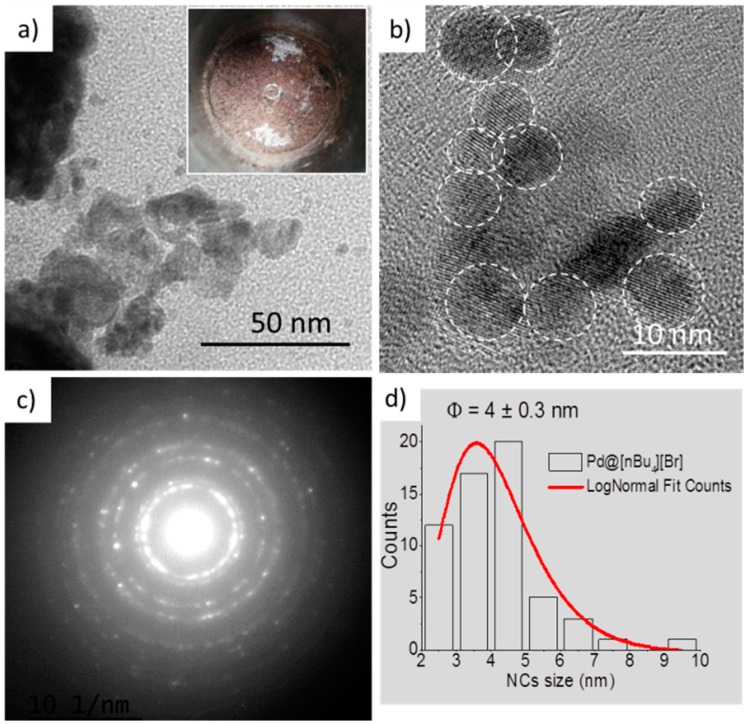
(**a**,**b**) TEM and HRTEM images of Pd@[*n*Bu_4_N][Br] NPs are presented; (**c**) High NPs crystallinity revealed by selected area diffraction pattern (SADP); (**d**) LogNormal size distribution of the NPs.

A high pressure/high temperature stirred stainless steel batch reactor (V_reactor_ = 60 mL) was used to synthesize for the first time the Pd NPs embedded in the solid matrix of quaternary ammonium salts: (1) tetrabutylammonium bromide, [*n*Bu_4_N][Br]; (2) tetraethylammonium bromide, [Et_4_N][Br]; (3) benzyltrimethylammonium bromide, [BnNMe_3_][Br]; (4) cetyltrimethylammonium bromide, [C_16_NMe_3_][Br]; (5) cetyltrimethylammonium bis(trifluoromethylsulfonyl)imide [C_16_NMe_3_][NTf_2_] and (6) cetyltrimethylammonium hexafluorophosphate, [C_16_NMe_3_][PF_6_]. Three bar of H_2_ as reducing agent were first loaded into the reactor containing the metal precursor (Pd(OAc)_2_) in powder form mixed with the solid OS to give 1 wt % metal loading. The synthesis reaction took place in a *sc*CO_2_ media, pressurized at 25 MPa, during 60 min at 100 °C, followed by fast depressurization and CO_2_ venting through the reactor (a few minutes more) at the end of the reaction in order to dry and clean the final material. A more detailed description of the synthesis approach is reported elsewhere [58]. The prepared final nanoparticles embedded in the solid matrix of ammonium salt, Pd@OS were recovered as dark powder (Figure 1a—inset) and used directly in the reaction of *N-*alkylation.

Microscopy images of the prepared nanocatalyst, Pd@[*n*Bu_4_N][Br], are presented in Figure 1. Using the *sc*CO_2_ approach the prepared Pd NPs are small but deceivingly more agglomerated than expected. An explanation could be due to the high reactivity of primary Pd nuclei obtained in supercritical media, already observed by our group [59,60]. Although [*n*Bu_4_N][Br], due to its structure, can act as electrostatic stabilizer preventing NPs growth and agglomeration, in the case of Pd, the electrostatic protection is not strong enough to overcome the agglomeration of the highly reactive NPs.

**Figure 2:**
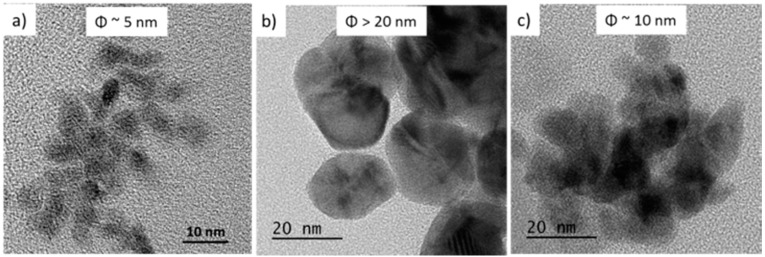
TEM images of Pd@OS NPs: (**a**) Pd@[BnNMe_3_][Br]; (**b**) Pd@[C_16_NMe_3_][Br] and (**c**) Pd@[C_16_NMe_3_][NTf_2_].

Very small and monodispersed NPs can also be obtained, as we have observed experimentally for other metals such as Ru, Ir, Pt [58]. The Pd NPs are however monocrystalline (Figure 1b,c) with a mean size of about 4 nm (Figure 1d). If this bulkier cation containing four symmetric alkyl chains is replaced by BnNMe_3_, less symmetrical and containing an aromatic ring, the Pd NPs size, morphology and chemical properties are affected. The obtained NPs are less aggregated, larger in size and less spherical (Figure 2a) leading eventually to different catalytic activity. It was assumed that the use of an OS with a long alkyl chain could stabilize the NPs more efficiently thus avoiding aggregation. With cetyltrimethylammonium bromide, slight improvement in monodispersity and an increase in size for Pd NPs were obtained (Figure 2b,c) with potential repercussions on NPs catalytic activity.

These Pd@OSs systems were then tested in alkylation of anilines using alcohols as alkylating agents. Noticeably, all obtained systems displayed interesting catalytic activities owing to the absence of NP surface modification during the material synthesis process. Indeed, unlike most liquid phase preparation of M(0) NP, surface partial oxidation or residual molecular solvent binding is avoided by the use of *sc*CO_2_. In all cases, OSs form a protective shell around the NPs allowing for shelf storage at room temperature in standard vials.

### 2.2. Optimisation of the N-Alkylation Reaction Conditions

A first screening of the catalytic system and reaction conditions was done on a model reaction between benzyl alcohol (**1a**) and aniline (**2a**). Two different simple palladium precursors were tested and only the nanoparticles generated from Pd(OAc)_2_ precursor in tetrabutylammonium bromide, labelled as Pd@[*n*Bu_4_N][Br], with 0.2 mol %, gave product **3aa** in 48% yield (Table 1, Entry 1). NPs generated from PdCl_2_ led to less active species, probably due to the passivation of the Pd surface by remaining chlorides.

By increasing the catalyst amount to 0.5 and 1 mol %, better yields of 71% and 78% (Table 1, Entries 2–3) were achieved. In situ nanoparticle generation provided the desired product in 71% yield, slightly lower than with the ex situ formed catalytic system (Table 1, Entry 4). Studying the ratio between benzyl alcohol and aniline, the results showed that the use of excess alcohol can be beneficial, leading to 86% yield. A 5 to 1 ratio gave only 85% yield (Table 1, Entries 5–6). In contrast, amine excess reduced the yield to 60%.

Many solvents were evaluated. Among those, ethylene glycol and diglyme had a negative effect, with 44% and 46% yields, respectively (Table 1, Entries 8–9). In the case of ethylene glycol, no competitive *N-*alkylation with solvent was observed. In the presence of toluene, an excellent yield of 97% was observed (Table 1, Entry 10). Mesitylene was less effective with 78% yield (Table 1, Entry 11). Using a more polar aromatic solvent, such as anisole, compound **3aa** was isolated in quantitative yield. The reaction could also take place in an aqueous medium, although leading to a mere 33% yield (Table 1, Entry 12).

Various Pd@OS, OS being chosen from the alkyl ammonium compound class, have been tested. With tetraethylammonium bromide [Et_4_N][Br] and benzyltrimethylammonium bromide [BnNMe_3_][Br], the generated nanoparticles seemed less active (28% and 43% yield, respectively, Table 1, Entries 13–14). In addition, the influence of the long alkyl chains of the cationic part and the counter-anion has been tested. Pd@[C_16_NMe_3_][Br], Pd@[C_16_NMe_3_][NTf_2_] and Pd@[C_16_NMe_3_][PF_6_] gave less promising results than those obtained with Pd@[*n*Bu_4_N][Br] (Table 1, Entries 16–18). Bromide and hexafluorophosphate are more effective than triflimidate counter-ions, mostly due to the different Pd NPs size observed. Noticeably, the catalytic properties could arise from the cumulative contribution of the structure and electronic properties of OS with the physicochemical properties (size, morphology, surface chemistry, organization) of Pd NPs.

### 2.3. N-Alkylation of Amines with Benzyl Alcohol Derivatives

Subsequent screening and optimization, Pd@[*n*Bu_4_N][Br] NPs system was found to be the best nanocatalyst. Therefore, this catalyst was further used to extend the scope of the “hydrogen-borrowing” methodology. Different amines and alcohols derivatives were tested in the previously optimised conditions (Table 2). Model reaction between benzyl alcohol (**1a**) and aniline (**2a**) provides *N-*benzylaniline (**3aa**) in 96% isolated yield (Table 2, Entry 1). Aniline *para* substitution with an alkyl group, R^2^ = Me or *t*Bu provides the desired amines **3ac** and **3ab** in good yields (95% and 91%, respectively; Table 2, Entries 2–3). In the presence of an electron donating group such as methoxy group, compound **3ad** is isolated in 91% yield (Table 2, Entry 4). An electron withdrawing group such as chlorine afforded product **3af** in good yield (88%, Table 2, Entry 5). Different aniline substitutions with fluorine in *para* and *meta* positions, also led to good yields of **3af** and **3ag** (Table 2, Entries 6–7). In the case of *ortho* substitution, no conversion took place, a phenomenon observed for all substituted products at this position. Polysubstituted anilines led to excellent yields. **3ah**, **3ai**, **3aj** are respectively isolated in 94%, 87% and 97% yields (Table 2, Entries 8–10). A strong electron withdrawing group slightly decreased the yield of target **3ak** to 76% (Table 2, Entry 11). The reaction is not limited to anilines. With linear primary amines such as benzylamine (**2m**) and hexylamine (**3an**), the reaction was effective, leading to **3am** and **3an** in 93% and 88% yields, respectively (Table 2, Entries 12–13). Secondary amines, such as morpholine (**2l**), provided compound **3al** in 52% yield (Table 2, Entry 14). Further heating at 160 °C improves the yield to 65%, but decomposition of the onium salt and morpholine became non-negligible.

Several benzylic alcohols were tested next (See NMR in Supplementary Materials). In the presence of electron donor groups such as 4-Me **1b** and 4-OMe **1c**, the reaction was effective with respective yields of 92% and 97% (Table 2, Entries 15–16). In the case of electron withdrawing groups, only the 4-Cl group provided the desired compound **3da** (50%, Table 2, Entry 17). In other cases, deactivated benzyl alcohol derivatives and aliphatic alcohols, no conversion was observed showing that the oxidation step is the limiting step. Furfuryl alcohol **1e** provides the *N-*alkylation **3ea** with a yield of 54% (Table 2, Entry 18).

### 2.4. Heterogeneous Catalysts for Cascade Oxidation/Imine Foration/Hydrogenation Leading to Benzylamine

**Figure 3:**
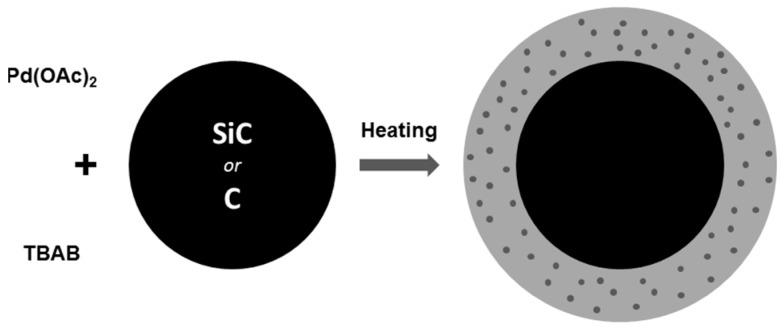
Preparation of Pd@TBAB@(Si)C.

In an attempt to provide an easily accessible heterogeneous catalyst, we prepared coated SiC and C with the previously described Pd@TBAB. The formation of the Pd NPs was directly performed in the presence of the solid material leading to Pd@TBAB@SiC and Pd@TBAB@C respectively (Figure 3). Depending on the TBAB/Support ratio, the material is obtained as beads coated with TBAB or a grey solid of Pd NP and matrix embedded in TBAB.

In short, silicon carbide turned out to be more efficient than the carbon black particle. This could be attributed to a better inertness of the support not interfering with the low palladium content. This is corroborated by the poor conversion obtained when a large excess of C is used (Table 3, Entry 4). The effect is more limited with silicon carbide, and with a same excess of support, a decent 59% yield is obtained after 24 h in anisole. Nonetheless, the best results were obtained with a Pd:TBAB:SiC ratio of 1:250:500 (Table 3, Entry 8) where both the activity (99% yield) and the form of the catalyst (grey powder) were appealing. When lowering the catalytic charge on the TBAB:SiC mixture, yield remained correct (Table 3, Entries 9–12), only with a 1:250:250 ratio were obtained quantitative yield. However in that case, catalyst is obtained as Pd NPs and SiC particles embedded in TBAB, and practically inconvenient for recycling tests.

Recycling experiments were performed using the carbon support, and unsurprisingly, the catalytic activity in the first cycle was deceiving. In that case, a complete loss of activity was observed after recovering the catalyst by filtration (Table 4, Entry 2).

This phenomenon, due to PdNP release in the reaction mixture was also observed using the 1:250/500 SiC catalysts but only after five cycles (Table 4, Entries 3–7). This is due to the relatively high solubility of TBAB in anisole, leading to leaching of Pd NPs into the reaction mixtures. In an attempt to limit this phenomenon, the solvent was switched to CO_2_ under supercritical conditions. TBAB is insoluble in *sc*CO_2_ but *sc*CO_2_ is soluble in the TBAB phase, favouring reagent/product exchange between *sc*CO_2_ phase and TBAB phase. While the reaction is inconclusive at 100 °C 80 bar, (Table 4, Entry 8), harsher reaction conditions led to a decent yield, which was maintained for three cycles owing to the insolubility of the catalytic active phase (Pd@TBAB) in the bulk solvent (*sc*CO_2_) (Table 4, Entries 9–11).

## 3. Experimental Section

### 3.1. General Information

GC-MS analysis were performed with a Agilent 7890A GC system (Agilent, Santa Clara, CA, USA) equipped with a J & W Scientific (Agilent) DB-1701 capillary column, a 5975C VL MSD with triple axis detector (EI) using the following program: 70 °C for 1 min then 20 °C·min^−1^ until 230 °C then 6 min at 230 °C. ^1^H-, ^13^C-NMR were recorded on 300 MHz Avance I spectrometers (Bruker, Billerica, MA, USA). The chemical shifts (δ) and coupling constants (*J*) are expressed in ppm and Hertz respectively. The following abbreviations were used to explain the multiplicities: s = singlet, d =doublet, m = multiplet.

### 3.2. Chemicals

Amines and alcohols were used without further purification, except for furfuryl alcohol and morpholine which were distillated over CaH_2_. All catalytic reactions were carried out under argon atmosphere. All chemicals were stored under argon. Silica gel (230–400 mesh) purchased from Merck (Merck KGaA, Darmstadt, Germany) was used for flash chromatography. Analytical TLC silica gel 60 F254 was used.

### 3.3. General Procedure

Pd@TBAB (1 mol % Pd in 250 mg of TBAB synthetized in *sc*CO_2_), alcohol (2 mmol, 2 eq), amine (1 mmol, 1 eq) and anisole (1 mL) were introduced into a sealed tube under argon. The reaction was then heated at 140 °C for 12 h. After cooling the reaction at room temperature, the mixture was filtrated on Celite and washed with Et_2_O and the filtrate was concentrated under vacuum. The crude oil was purified by column chromatography.

### 3.4. Analyses

*N-Phenylbenzenemethanamine* (**3aa**). CAS: 103-32-2; white solid; Yield: 96%. ^1^H-NMR (300 MHz, CDCl_3_, 25 °C) δ 7.42–7.27 (m, 5H), 7.24–7.14 (m, 2H), 6.78–6.69 (m, 1H), 6.66 (dd, *J* = 8.6, 1.0 Hz, 2H), 4.35 (s, 2H), 4.04 (bs, 1H) ^13^C-NMR (75 MHz, CDCl_3_, 25 °C) δ 148.0, 139.4, 129.4 (2C), 128.8 (2C), 127.8 (2C), 127.4, 118.0, 113.2 (2C), 48.6 MS *(m/z)* 183 (78), 106 (18), 91 (100), 77 (20), 65 (14), 51 (8).

*N-(4-Methylphenyl)-benzenemethanamine* (**3ab**). CAS: 5405-15-2; Pale yellow oil; Yield: 95%. ^1^H-NMR (300 MHz, CDCl_3_, 25 °C) δ 7.42–7.27 (m, 5H), 6.99 (d, *J* = 7.0 Hz, 2H), 6.57 (d, *J* = 7.0 Hz, 2H), 4.31 (s, 2H), 3.90 (bs, 1H), 2.24 (s, 3H) ^13^C-NMR (75 MHz, CDCl_3_, 25 °C) δ 145.1, 139.1, 130.2, 129.9 (2C), 128.7 (2C), 127.9 (2C), 127.4, 113.9 (2C), 49.3, 20.6 MS *(m/z)* 197 (74), 120 (21), 91 (100), 77 (10), 65 (14), 51 (4).

*N-(4-tert-Butylphenyl)-benzenemethanamine* (**3ac**). CAS : 255835-93-9; White solid; Yield: 91%. ^1^H-NMR (300 MHz, CDCl_3_, 25 °C) δ 7.49–7.37 (m, 4H), 7.38–7.31 (m, 2H), 7.19–7.10 (m, 1H), 6.80–6.68 (m, 2H), 4.45 (s, 2H), 4.36 (s, 1H), 1.48 (s, 9H) ^13^C-NMR (75 MHz, CDCl_3_, 25 °C) δ 165.8, 162.6, 149.9, 138.8, 130.5, 130.4, 128.9 (2C), 127.6 (2C), 127.5, 108.9, 104.4, 104.1, 99.9, 99.6, 48.4 MS *(m/z)* 239 (46), 224 (28), 196 (5), 132 (9), 120 (8), 106 (8), 91 (100), 77 (6), 56 (8), 51 (2).

*N-(4-Methoxyphenyl)-benzenemethanamine* (**3ad**). CAS: 17377-95-6; Pale yellow oil; Yield: 91%. ^1^H-NMR (300 MHz, CDCl_3_, 25 °C) δ 7.40 (d, *J* = 7.3 Hz, 2H), 7.30 (t, *J* = 6.3 Hz, 2H), 7.00 (dd, *J* = 5.3, 2.5 Hz, 2H), 6.83 (d, *J* = 5.7 Hz, 1H), 6.74 (d, *J* = 7.6 Hz, 2H), 4.35 (s, 2H), 4.02 (bs, 1H), 3.90 (s, 3H) ^13^C-NMR (75 MHz, CDCl_3_, 25 °C) δ 158.9, 148.3, 131.5, 129.3 (2C), 129.3 (2C), 117.6, 114.1 (2C), 112.9 (2C), 55.4, 47.9 MS *(m/z)* 213 (100), 198 (9), 168 (5), 136 (9), 122 (97), 91 (89), 77 (6), 65 (14), 51 (4).

*N-(4-Chlorophenyl)-benzenemethanamine* (**3ae**). CAS: 2948-37-0; Pale yellow oil; Yield: 88%. ^1^H-NMR (300 MHz, CDCl_3_, 25 °C) δ 7.35 (d, *J* = 4.5 Hz, 2H), 7.34–7.25 (m, 3H), 7.11 (d, *J* = 8.9 Hz, 2H), 6.55 (d, *J* = 8.9 Hz, 2H), 4.31 (s, 2H), 4.07 (bs, 1H). ^13^C-NMR (75 MHz, CDCl_3_, 25 °C) 145.3, 138.0, 129.28 (2C), 128.9 (2C), 127.9 (2C), 127.6, 123.7, 115.2 (2C), 49.3. MS (*m/z*) 217 (40), 140 (7), 111 (6), 91 (100), 65 (9), 51 (3).

*N-(4-Fluorophenyl)-benzenemethanamine* (**3af**). CAS: 370-77-4; Pale yellow oil; Yield: 92 %. ^1^H-NMR (300 MHz, CDCl_3_, 25 °C) δ 7.43–7.27 (m, 5H), 6.97–6.84 (m, 2H), 6.63–6.53 (m, 2H), 4.31 (s, 2H), 3.94 (s, 1H). ^13^C-NMR (75 MHz, CDCl_3_, 25 °C) δ 157.8 (s), 154.7 (s), 143.8 (s), 138.7 (s), 128.7 (s), 127.6 (*J_CF_* = 18.1 Hz), 115.9 (s), 115.6 (s), 114.3 (*J_CF_* = 7.5 Hz), 77.5 (s), 77.1 (s), 76.7 (s), 49.3 (s). MS *(m/z)* 201 (53), 124 (9), 91 (100), 65 (10), 51 (3).

*N-(3-Fluorophenyl)-benzenemethanamine* (**3ag**). CAS: 123330-53-0; Pale yellow oil; Yield: 87%. ^1^H-NMR (300 MHz, CDCl_3_, 25 °C) δ 7.37–7.36 (m, 4H), 7.32–7.28 (m, 1H), 7.12–7.08 (m, 1H), 6.41–6.39 (m, 2H), 6.34–6.31(m, 1H), 4.32 (s, 2H), 4.18 (s, 1H). ^13^C-NMR (75 MHz, CDCl_3_, 25 °C) δ 164.4 (*J*_CF_ = 241 Hz), 150.1 (*J*_CF_ = 11Hz), 139.0, 130.5 (*J*_CF_ = 10 Hz), 129.0, 127.7, 127.6, 109.0, 104.2 (*J*_CF_ = 21 Hz), 99.7 (*J*_CF_ = 25 Hz), 48.4. MS (*m/z*) 201 (52), 124 (8), 91 (100), 65 (16), 51 (5). 

*N-(3,4-Dichlorophenyl)-benzenemethanamine* (**3ah**). CAS: 51597-75-2; Pale yellow oil; Yield: 94%. ^1^H-NMR (300 MHz, CDCl_3_, 25 °C) δ 7.44–7.26 (m, 1H), 7.18 (d, *J* = 8.7 Hz, 1H), 6.71 (d, *J* = 2.7 Hz, 1H), 6.46 (dd, *J* = 8.7, 2.8 Hz, 1H), 4.29 (s, 1H), 4.23 (s, 1H). ^13^C-NMR (75 MHz, CDCl_3_, 25 °C) 147.6, 138.4, 132.9, 130.7, 128.9 (2C), 127.7, 127.5 (2C), 120.2, 114.1, 112.7, 48.3. MS (*m/z*) 251 (27), 174 (4), 145 (4), 91 (100), 65 (8), 51 (2). 

*N-(3,5-Dichlorophenyl)-benzenemethanamine* (**3ai**). CAS: 65089-00-1; Pale yellow oil; Yield: 87%. ^1^H-NMR (300 MHz, CDCl_3_, 25 °C) δ 7.47 – 7.32 (m, 5H), 6.72 (t, *J* = 1.7 Hz, 1H), 6.53 (d, *J* = 1.7 Hz, 2H), 4.35 (s, 1H), 4.33 (s, 2H). ^13^C-NMR (75 MHz, CDCl_3_, 25 °C) δ 140.8, 136.1, 129.6, 128.6 (2C), 127.6, 126.9 (2C), 123.2, 120.2, 113.4, 48.2. MS (*m/z*) 251 (27), 174 (4), 145 (4), 91 (100), 65 (8), 51 (2).

*N-(3-Chloro-4-fluorophenyl)-benzenemethanamine* (**3aj**). CAS: 131776-32-4; Pale yellow oil; Yield: 97%. ^1^H-NMR (300 MHz, CDCl_3_, 25 °C) δ 7.36–7.26 (m, 4H), 7.24–7.20 (m, 1H), 7.06 (t, *J* = 9.2 Hz,1H), 6.63 (dd, *J* = 2.8, 6.4 Hz, 1H), 6.51 (dt, *J* = 3.2, 9.2 Hz, 1H), 6.43 (t, *J* = 5.8 Hz, 1H), 4.22 (s, 2H, *J* = 6.0 Hz). ^13^C-NMR (75 MHz, CDCl_3_, 25 °C) 153.2, 146.2, 146.1, 138.7, 128.9, 128.7, 127.8, 127.6, 127.6, 117.0, 113.9, 112.1, 48.0. MS (*m/z*) 235 (32), 158 (5), 129 (6), 91 (100), 65 (8), 51 (2).

*N-(4-(Trifluoromethyl)phenyl)-benzenemethanamine* (**3ak**). CAS: 405-81-2; Pale yellow oil; Yield: 76%. ^1^H-NMR (300 MHz, CDCl_3_, 25 °C) δ 7.49–7.16 (m, 6H), 7.02–6.90 (m, 1H), 6.86 (m, 1H), 6.76 (dd, *J* = 8.2, 2.3 Hz, 1H), 4.35 (s, 2H), 1.54 (s, 1H). ^13^C-NMR (75 MHz, CDCl_3_, 25 °C) δ 150.6, 138.6, 128.9, 127.6, 127.5, 126.8, 123.7, 119.2 (q, *J*_CF_ = 32.5 Hz), 48.0. MS (*m/z*) 251 (42), 174 (7), 145 (11), 91 (100), 65 (8), 51 (2).

*N-(Phenylmethyl)-benzenemethanamine* (**3am**). CAS: 103-49-1; Pale yellow oil; Yield: 93%. ^1^H-NMR (300 MHz, CDCl_3_, 25 °C) δ 7.61–7.29 (m, 10H), 3.93 (s, 4H), 1.76 (s, 1H). ^13^C-NMR (75 MHz, CDCl_3_, 25 °C) δ 140.4 (2C), 128.4 (4C), 128.2 (4C), 127.0 (2C), 53.2 (2C). MS *(m/z)* 196 (14), 120 (8), 106 (57), 91 (100), 77 (6), 65 (14), 51 (5).

*N-Hexylbenzenemethanamine* (**3an**). CAS: 25468-44-4; Pale yellow oil; Yield: 88%. ^1^H-NMR (300 MHz, CDCl_3_, 25 °C) δ 7.47–7.22 (m, 5H), 3.84 (s, 2H), 2.67 (t, *J* = 9.3 Hz, 2H), 1.64–1.29 (m, 6H), 0.94 (t, *J* = 9.3 Hz, 2H). ^13^C-NMR (75 MHz, CDCl_3_, 25 °C) δ 140.7, 128.5 (2C), 128.2 (2C), 126.9, 54.2, 49.7, 31.9, 30.2, 27.2, 22.7, 14.2. MS (*m/z*) 191(2), 120 (65), 106 (8), 91 (100), 65 (6).

*4-(Phenylmethyl)-morpholine* (**3al**). CAS: 10316-00-4; yellow oil; Yield: 52%. ^1^H-NMR (300 MHz, CDCl_3_, 25 °C), 7.45–7.28 (m, 5H), 4.68 (s, 2H), 2.1–2.0 (m, 8H). ^13^C-NMR (75 MHz, CDCl_3_, 25 °C), 140.5, 129.4, 128.1, 126.9, 66.3, 64.9, 60.4. MS (*m/z*) 177 (30), 146 (31), 91 (100), 86 (17), 65 (11), 56 (6). 

*4-Methyl-N-phenylbenzenemethanamine* (**3ba**). CAS: 15818-64-1; Pale yellow oil; Yield: 92%. ^1^H-NMR (300 MHz, CDCl_3_, 25 °C) δ 7.31 (d, *J* = 7.9 Hz, 1H), 7.27–7.15 (m, 2H), 6.82–6.73 (m, 1H), 6.73–6.65 (m, 1H), 4.33 (s, 1H), 4.11 (s, 1H), 2.40 (s, 1H). ^13^C-NMR (75 MHz, CDCl_3_, 25 °C) δ 148.2, 136.9, 136.4, 129.3 (2C), 129.2 (2C), 127.6 (2C), 117.5, 113.9, 112.9, 48.1, 21.2. MS (*m/z*) 197 (48), 105 (100), 77 (18), 65 (4), 51 (4). 

*4-Methoxy-N-phenylbenzenemethanamine* (**3ca**). CAS: 3526-43-0; Pale yellow oil; Yield: 97%. ^1^H-NMR (300 MHz, CDCl_3_, 25 °C) δ 7.40 (d, J = 7.3 Hz, 2H), 7.30 (t, *J* = 6.3 Hz, 2H), 7.00 (dd, *J* = 5.3, 2.5 Hz, 2H), 6.83 (d, *J* = 5.7 Hz, 1H), 6.74 (d, *J* = 7.6 Hz, 2H), 4.35 (s, 2H), 4.02 (s, 1H), 3.90 (s, 3H). ^13^C-NMR (75 MHz, CDCl_3_, 25 °C) δ 159.0, 148.3, 131.5, 129.3 (2C), 128.9 (2C), 117.6, 114.1 (2C), 112.9 (2C), 55.4, 47.9. MS (*m/z*) 213 (20), 121 (100), 77 (11), 51 (3).

*4-Chloro-N-phenylbenzenemethanamine* (**3da**). CAS: 4750-61-2; Pale yellow oil; Yield : 50% ^1^H-NMR (300 MHz, CDCl_3_, 25 °C) δ 7.54–7.31 (m, 4H), 7.31–7.17 (m, 2H), 6.80 (td, *J* = 7.3, 1.0 Hz, 1H), 6.67 (dd, *J* = 7.8, 0.7 Hz, 2H), 4.37 (s, 2H), 4.12 (s, 1H). ^13^C-NMR (75 MHz, CDCl_3_, 25 °C) δ 147.9, 138.1, 133.0, 129.4 (2C), 128.9 (2C), 128.8 (2C), 117.9, 113.0 (2C), 47.7. MS (*m/z*) 214 (100), 104 (14), 89 (10), 77 (63), 51 (18).

*N-Phenyl-2-furanmethanamine* (**3ea**). CAS: 4439-56-9; Pale yellow oil; Yield: 54%. ^1^H-NMR (300 MHz, CDCl_3_, 25 °C) δ 7.48–7.44 (m, 1H), 7.34–7.22 (m, 2H), 6.83 (td, *J* = 7.3, 1.0 Hz, 1H), 6.76 (dd, *J* = 7.7, 0.9 Hz, 2H), 6.40 (dd, *J* = 3.0, 1.7 Hz, 1H), 6.37–6.28 (m, 1H), 4.39 (s, 2H), 4.08 (s, 1H). ^13^C-NMR (75 MHz, CDCl_3_, 25 °C) δ 152.8, 148.7, 142.0, 129.3 (2C), 118.1, 113.3 (2C), 110.4, 107.0, 41.5. MS (*m/z*) 173 (48), 81 (100), 77 (12), 65 (6), 53 (7).

## 4. Conclusions

Owing to the key combination of onium salts and *sc*CO_2_, a flexible, robust, solvent-free preparation of Pd NPs has been done. In contrast with many other methods, it provided a ready-to-use, stable, storable powder containing Pd NPs with unmodified surface leading to high catalytic activity. The *N-*alkylation of amines with benzylic alcohols was optimized in the absence of base or other additives classically used in this case, and generated only water as by-product in the presence of nanocatalysts. Recycling of Pd NP embedded in OS coated on SiC has yet to be improved to achieve a high conversion while ensuring a low leaching level. Supercitical millifluidics is currently under study to obtain a relevant continuous production system for benzylation of amines.

## Figures and Tables

**Table 1:**
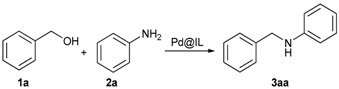
Optimization of *N-*alkylation of aniline with benzyl alcohol ^1^.

Entry	Ratio	OS	Pd@IL	Solvent	Yield (%) ^2^
	1a:2a		(mol %)		
1	1:1	[*n*Bu_4_N][Br]	0.2	-	48
2	1:1	[*n*Bu_4_N][Br]	0.5	-	71
3	1:1	[*n*Bu_4_N][Br]	1	-	78
4	1:1	[*n*Bu_4_N][Br]	1	-	71
5	2:1	[*n*Bu_4_N][Br]	1	-	86
6	5:1	[*n*Bu_4_N][Br]	1	-	85
7	1:2	[*n*Bu_4_N][Br]	1	-	60
8	2:1	[*n*Bu_4_N][Br]	1	EG ^3^	44
9	2:1	[*n*Bu_4_N][Br]	1	diglyme	46
10	2:1	[*n*Bu_4_N][Br]	1	toluene	97
11	2:1	[*n*Bu_4_N][Br]	1	mesitylene	78
12	2:1	[*n*Bu_4_N][Br]	1	anisole	>99
13	2:1	[*n*Bu_4_N][Br]	1	water	33
14	2:1	[Et_4_N][Br]	1	toluene	28
15	2:1	[BnNMe_3_][Br]	1	toluene	43
16	2:1	[C_16_NMe_3_][Br]	1	toluene	63
17	2:1	[C_16_NMe_3_][NTf_2_]	1	toluene	51
18	2:1	[C_16_NMe_3_][PF_6_]	1	toluene	62

^1^ General conditions: sealed tube, Ar, limiting reagent (1 mmol), solvent (1 mL), 140 °C, 24 h. ^2^ GC yields determined by reference to an internal standard. ^3^ EG: ethylene glycol.

**Table 2:**
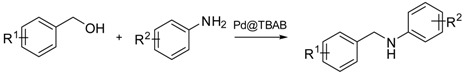
*N-*alkylation of amines with alcohols ^1^.

Entry	Alcohol 1	Amine 2	Product	Yield (%) ^2^
	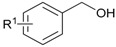	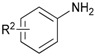	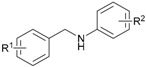	
1	**1a** (R^1^ = H)	**2a** (R^2^ = H)	**3aa**	96
2	**1a**	**2b** (R^2^ = 4-Me)	**3ab**	95
3	**1a**	**2c** (R^2^ = 4-tBu)	**3ac**	91
4	**1a**	**2d** (R^2^ = 4-OMe)	**3ad**	91
5	**1a**	**2e** (R^2^ = 4-Cl)	**3ae**	88
6	**1a**	**2f** (R^2^ = 4-F)	**3af**	92
7	**1a**	**2g** (R^2^ = 3-F)	**3ag**	87
8	**1a**	**2h** (R^2^ = 3,4-Cl)	**3ah**	94
9	**1a**	**2i** (R^2^ = 3,5-Cl)	**3ai**	87
10	**1a**	**2j** (R^2^ = 3-Cl,4-F)	**3aj**	97
11	**1a**	**2k** (R^2^ = 4-CF3)	**3ak**	76
12	**1a**	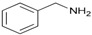 **2m**	**3am**	93
13	**1a**	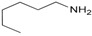 **2n**	**3an**	88
14	**1a**	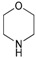 **2l**	**3al**	52
15	**1b** (R^1^ = 4-Me)	**2a**	**3ba**	92
16	**1c** (R^1 ^= 4-OMe)	**2a**	**3ca**	97
17	**1d** (R^1^ = 4-Cl)	**2a**	**3da**	50
18	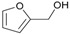 **1e**	**2a**	**3ea**	54

^1^ Pd@[*n*Bu_4_N][Br] (1% in Pd), **1** (2 mmol), **2** (1 mmol), anisole (1 mL), 140 °C, 24 h. ^2^ isolated yield.

**Table 3:**
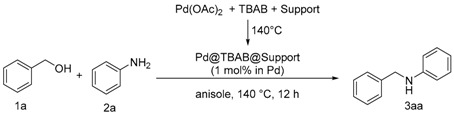
*N-*Alkylation of amines with alcohols using a heterogeneous catalyst ^1^.

Entry	Pd:TBAB:Support Ratio	Support	Yield (%) ^2^
1	1:250:250	C	60
2	1:250:500	C	35
3	1:250:1000	C	36
4	1:250:2000	C	9
5	1:500:1000	C	39
6	1:1000:1000	C	46
7	1:250:250	SiC	99
8	1:250:500	SiC	99
9	1:250:1000	SiC	73
10	1:250:2000	SiC	59
11	1:500:1000	SiC	77
12	1:1000:1000	SiC	80

^1^ Pd@[*n*Bu_4_N][Br]@M (1% in Pd), **1** (2 mmol), **2** (1 mmol), anisole (1 mL), 140 °C, 24 h. ^2^ isolated yield.

**Table 4:**
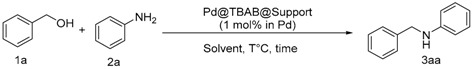
Recycling of heterogeneous catalyst ^1^.

Entry	Run	Support		Time	Yield (%) ^2^
1	1	C (1:250:250)	anisole ^3^	4 h	60
2	2	C (1:250:250)	anisole ^3^	4 h	0
3	1	SiC (1:250:500)	anisole ^3^	4 h	99
4	2	SiC (1:250:500)	anisole ^3^	4 h	98
5	3	SiC (1:250:500)	anisole ^3^	4 h	85
6	4	SiC (1:250:500)	anisole ^3^	4 h	60
7	5	SiC (1:250:500)	anisole ^3^	4 h	0
8	1	SiC (1:250:500)	*sc*CO_2_^4^	4 h	<5
9	1	SiC (1:250:500)	*sc*CO_2_^5^	6 h	51
10	2	SiC (1:250:500)	*sc*CO_2_^5^	6 h	50
11	3	SiC (1:250:500)	*sc*CO_2_^5^	6 h	50

^1^ Pd@[*n*Bu_4_N][Br]@M (1% in Pd), **1** (2mmol), **2** (1 mmol); ^2^ isolated yield; ^3^ anisole (1 mL), 140 °C; ^4^ CO_2_, 100 °C, 80 bar; ^5^ CO_2_, 140 °C, 100 bar.

## Supplementary Materials

Supplementary materials can be accessed at: http://www.mdpi.com/1420-3049/21/8/1042/s1.
