# Supplementary material for: Pd@[nBu4][Br] as a Simple Catalytic System for N-Alkylation Reactions with Alcohols
Source: Molecules. 2016 Aug 10;21(8):1042. doi: 10.3390/molecules21081042 (PMC6273040; doi:10.3390/molecules21081042)
Supplement: Supplementary file 1 [file molecules-21-01042-s001.pdf]

Supplementary Materials: Pd@[*n*Bu4][Br] as Simple Catalytic System for *N*-Alkylation Reaction with Alcohols

Bastien Cacciuttolo, Oana Pascu, Cyril Aymonier and Mathieu Pucheault

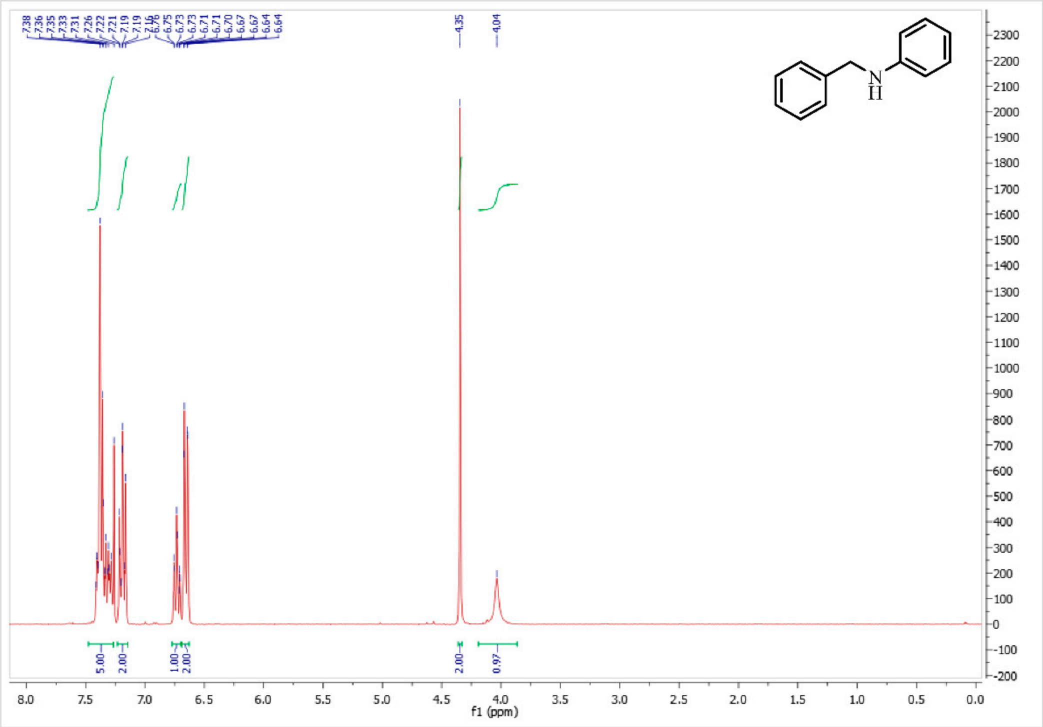

Figure S1. 3aa, ^{1}H-NMR.

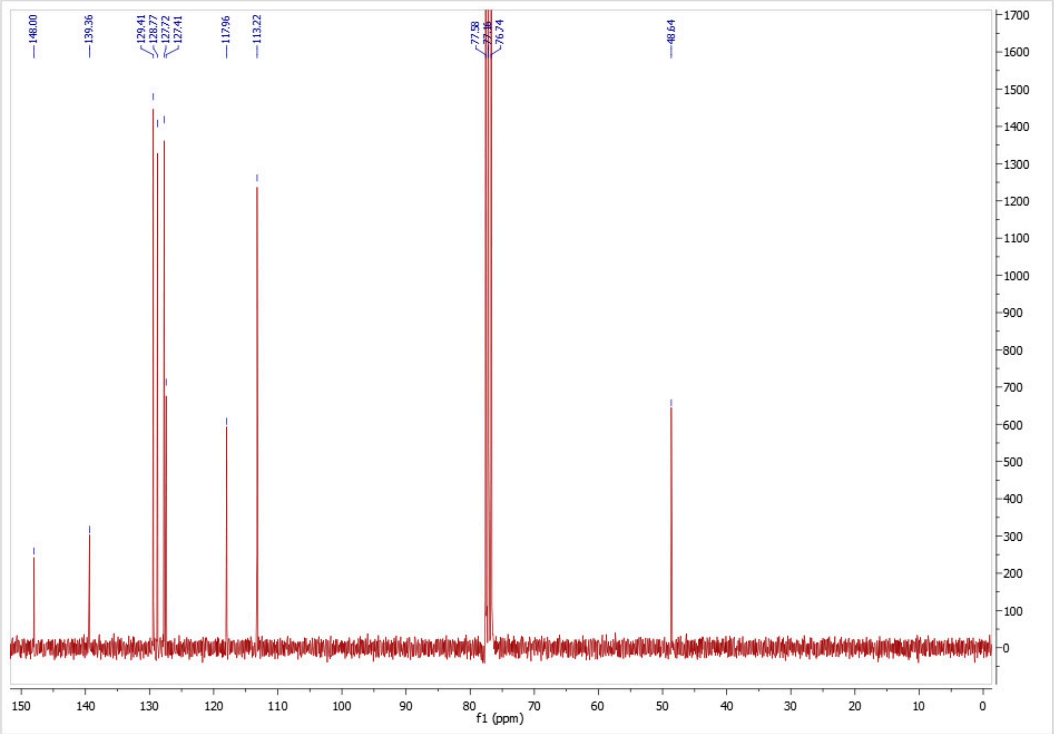

Figure S2. 3aa, ^{13}C-NMR.

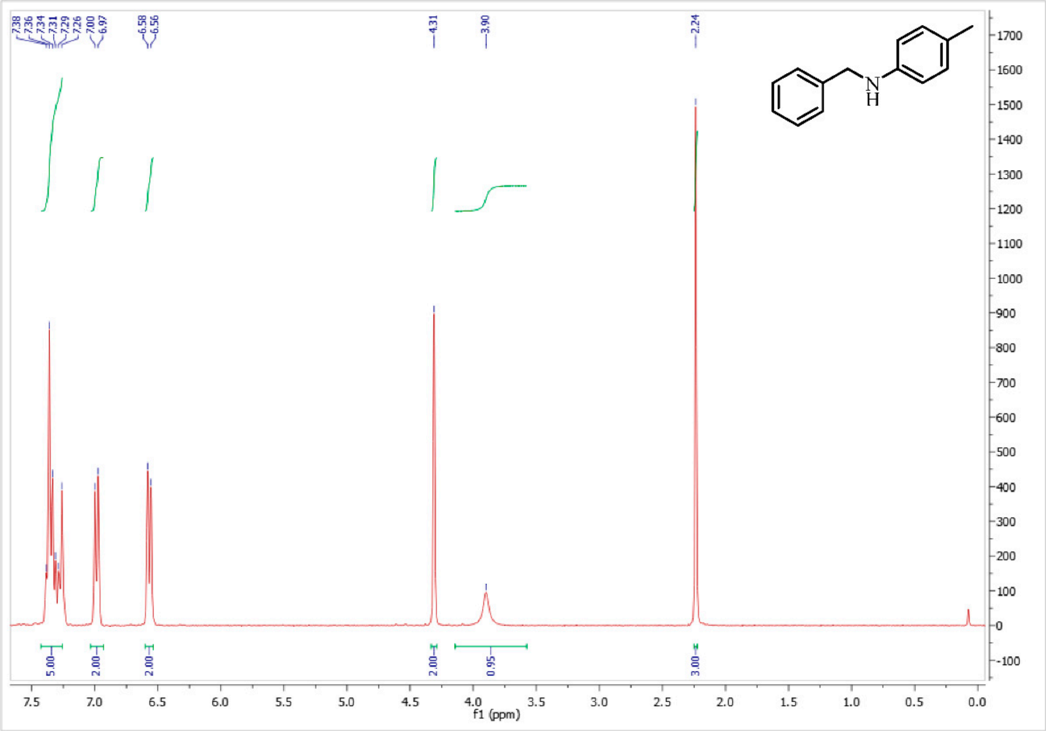

Figure S3. 3ab, ^{1}H-NMR.

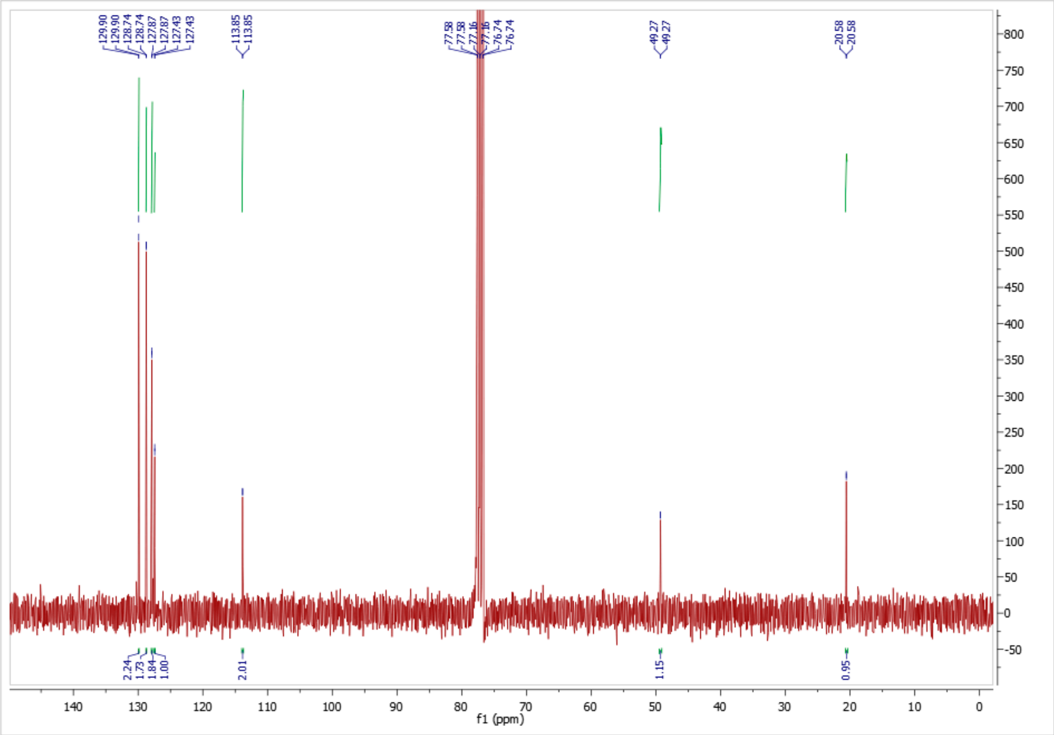

Figure S4. 3ab, ^{13}C-NMR.

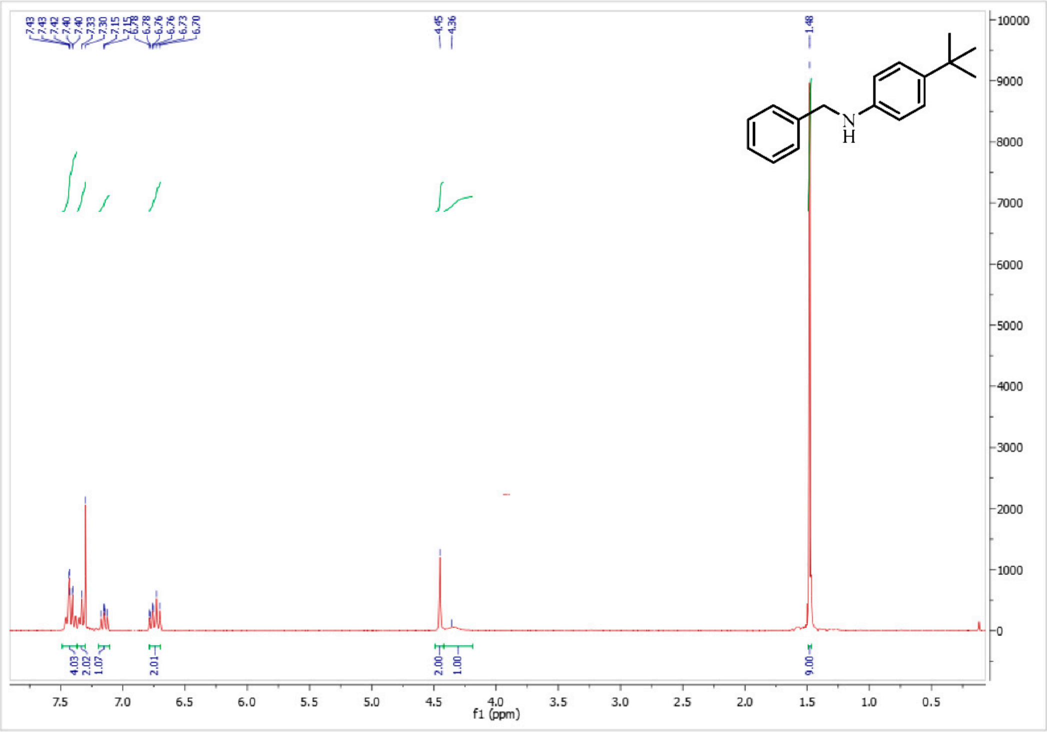

Figure S5. 3ac, ¹H-NMR.

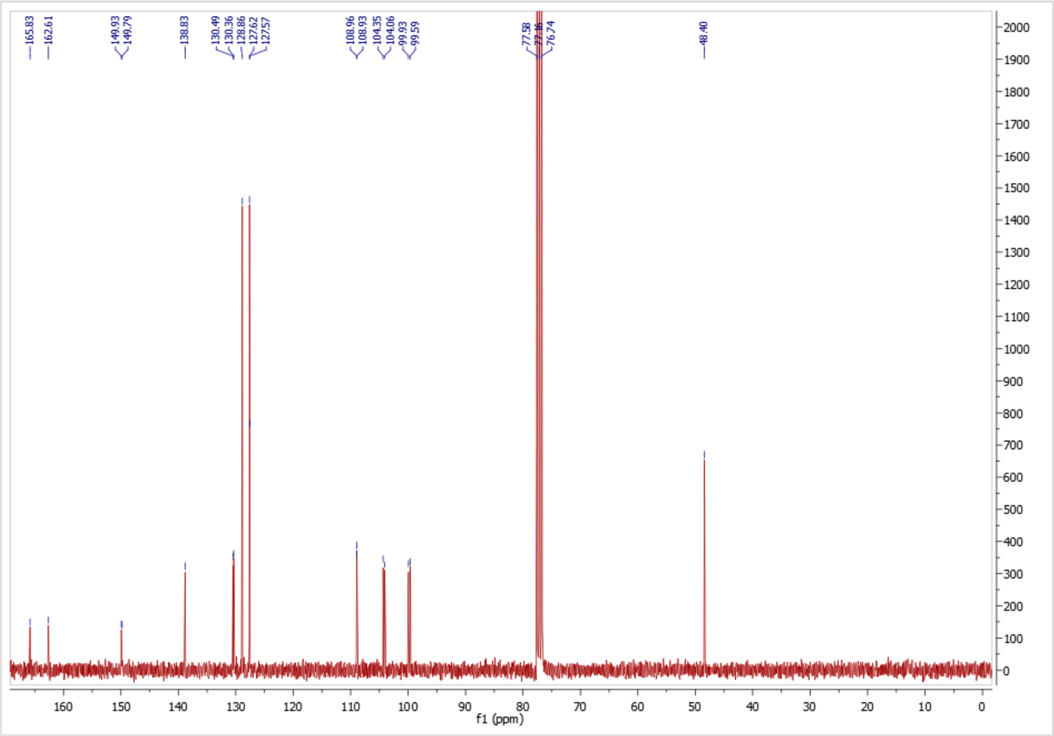

Figure S6. 3ac, ¹³C-NMR.

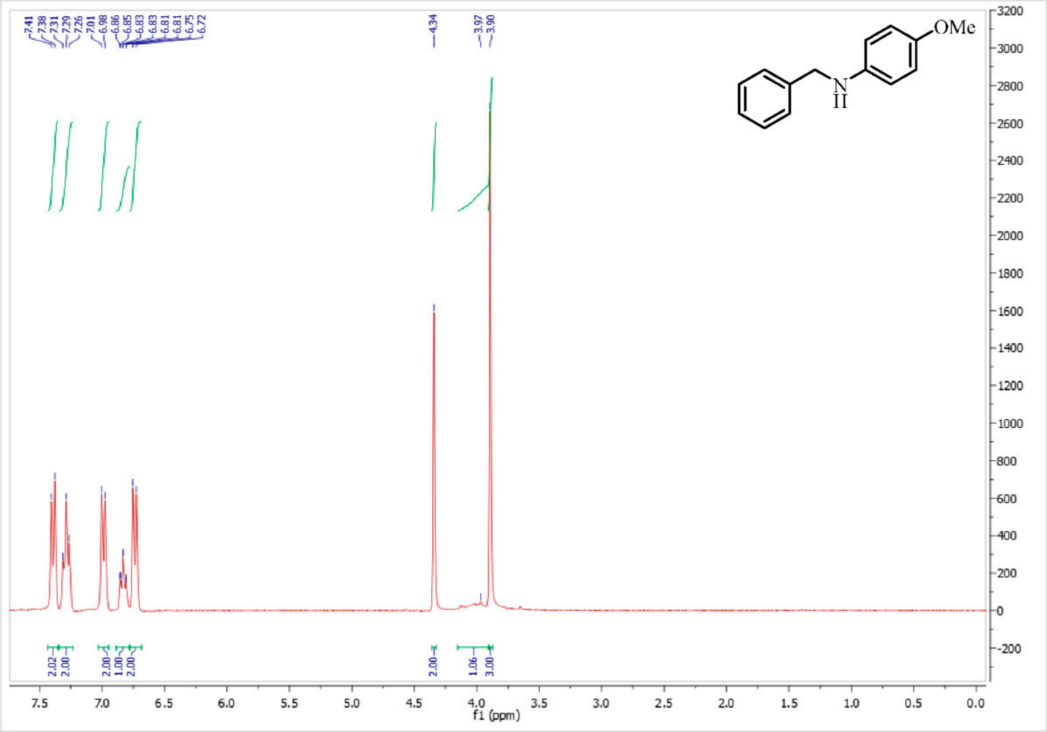

Figure S7. 3ad, ^{1}H-NMR.

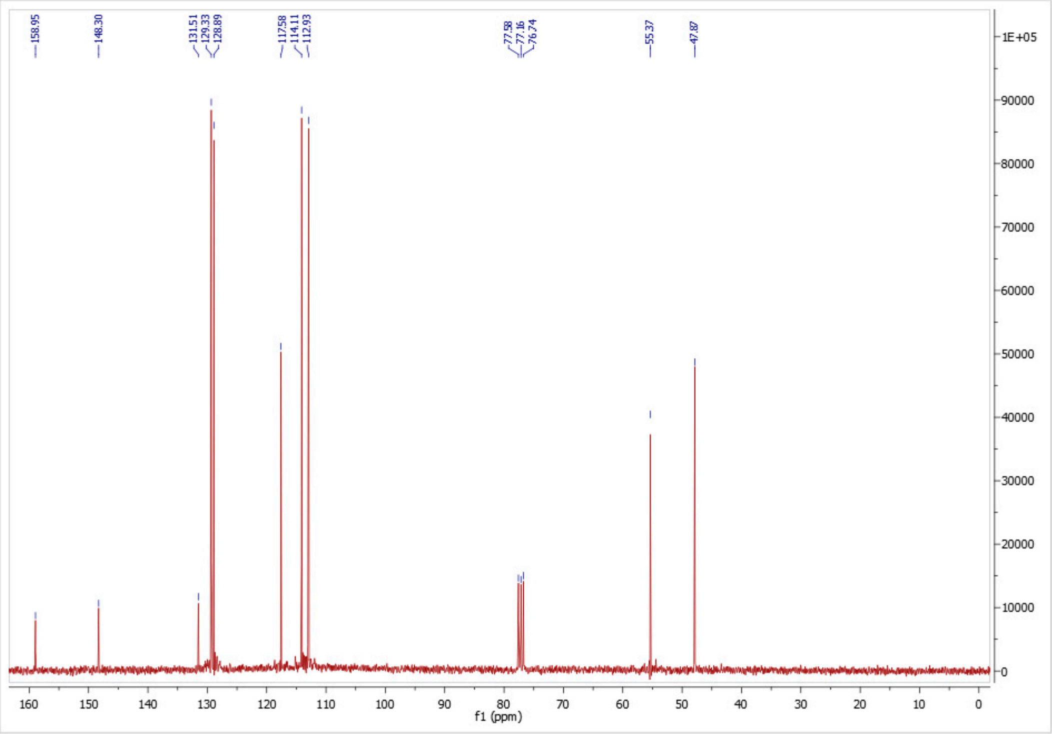

Figure S8. 3ad, ^{13}C-NMR.

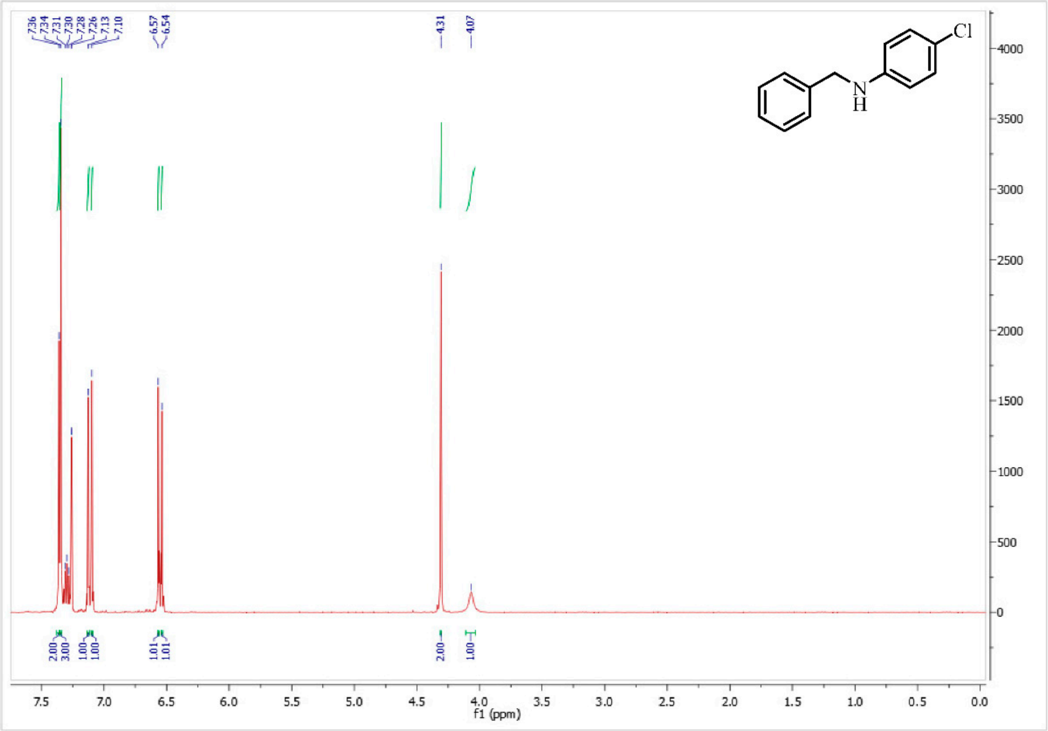

Figure S9. 3ae, ¹H-NMR.

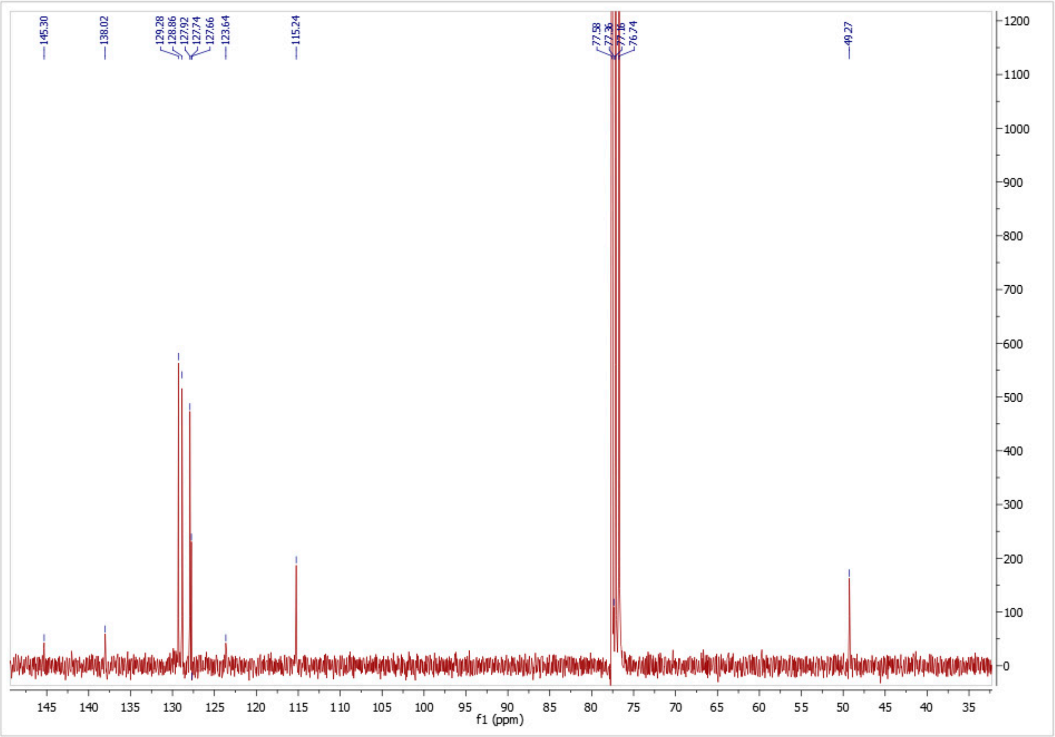

Figure S10. 3ae, ¹³C-NMR.

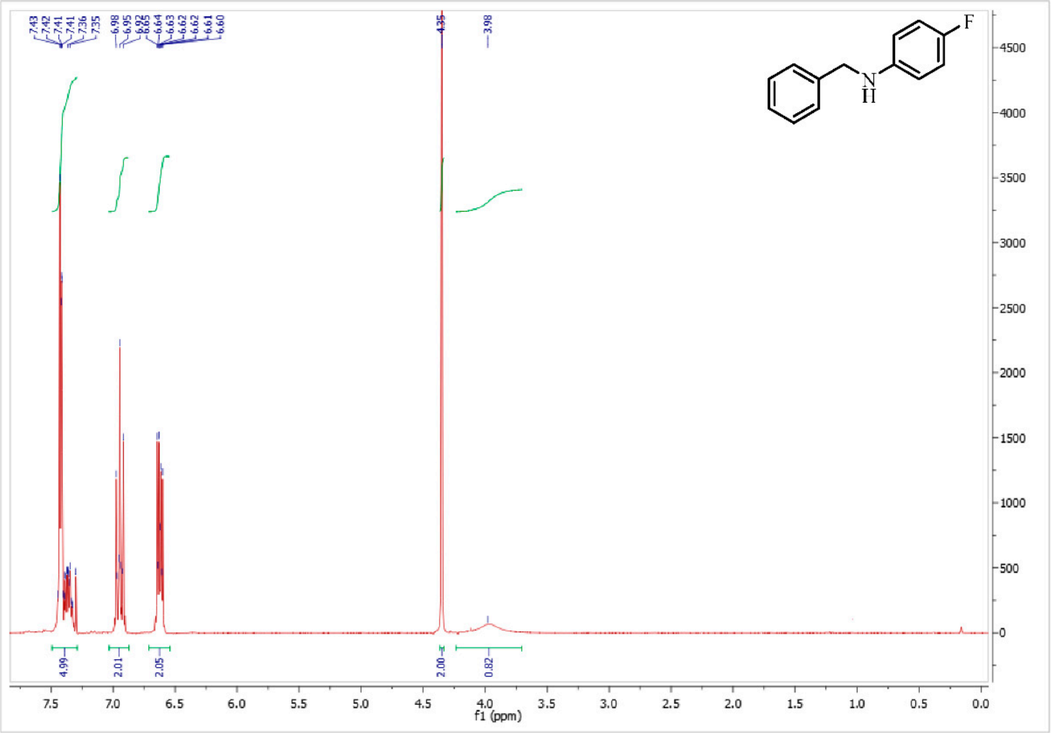

Figure S11. 3af, ^{1}H-NMR.

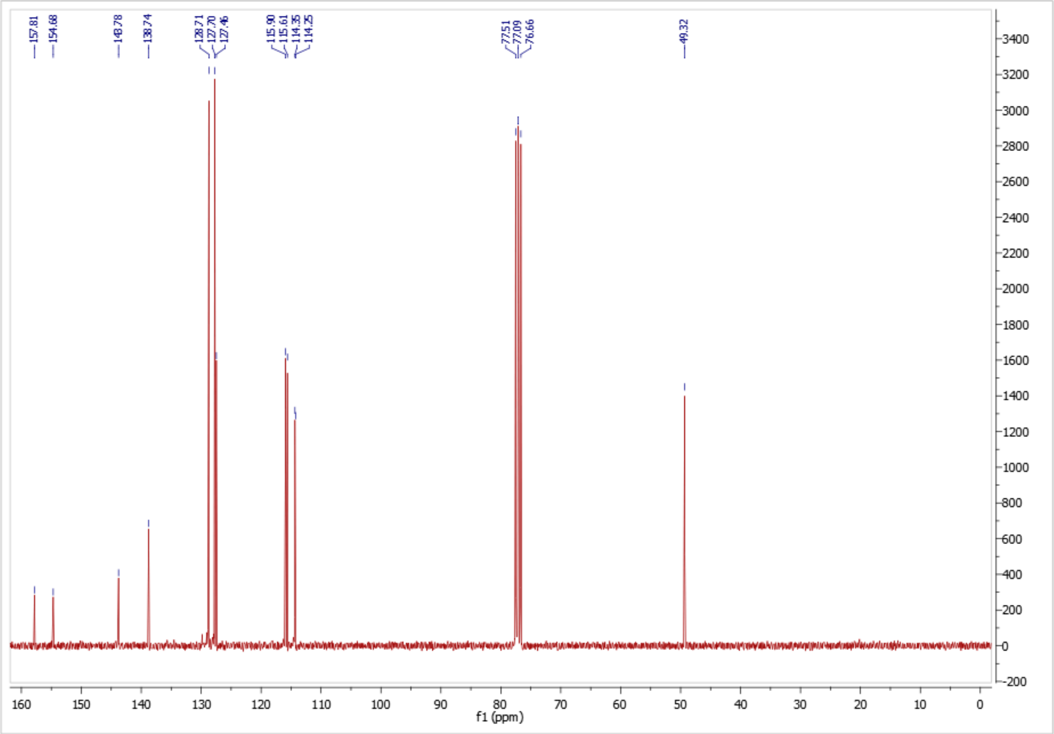

Figure S12. 3af, ^{13}C-NMR.

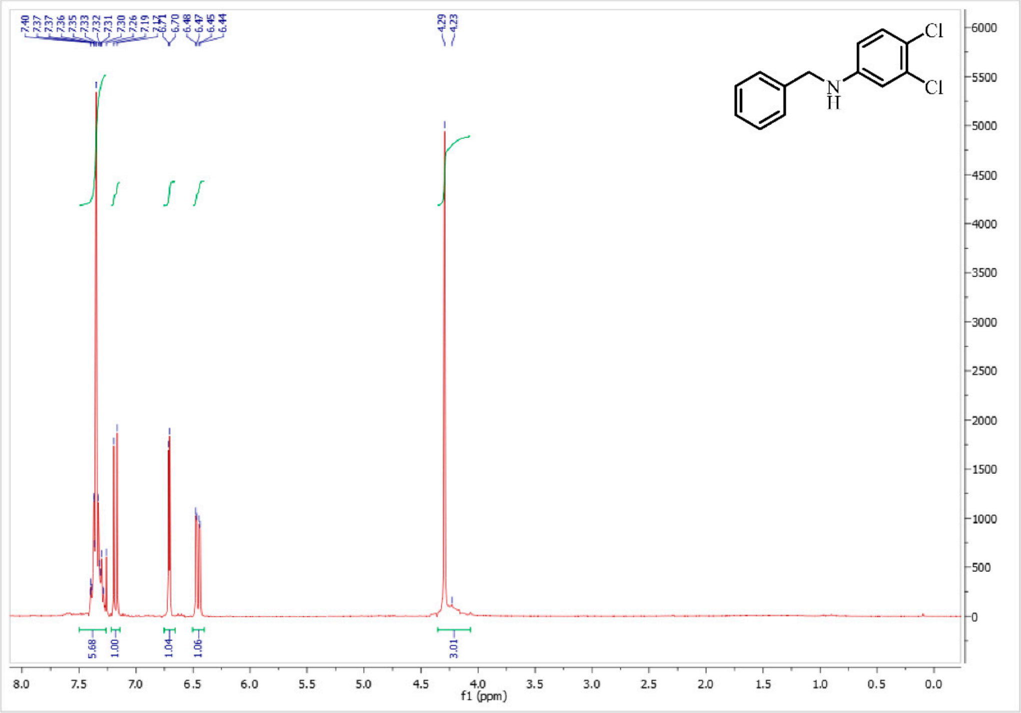

Figure S13. 3ah, ¹H-NMR.

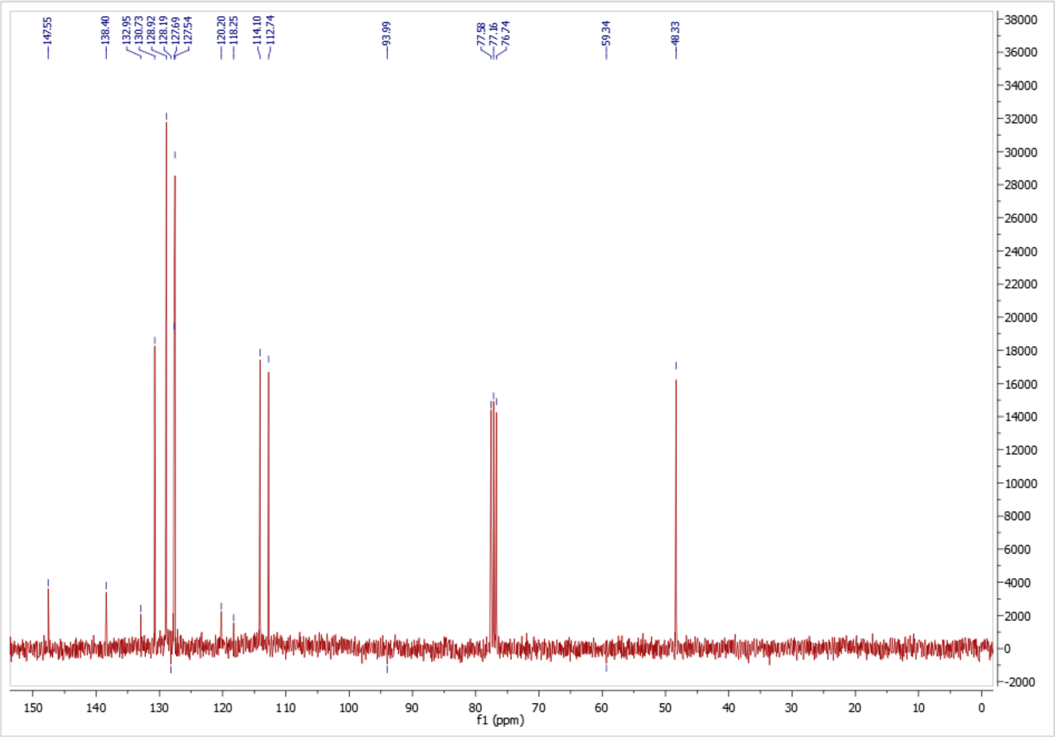

Figure S14. 3ah, ¹³C-NMR.

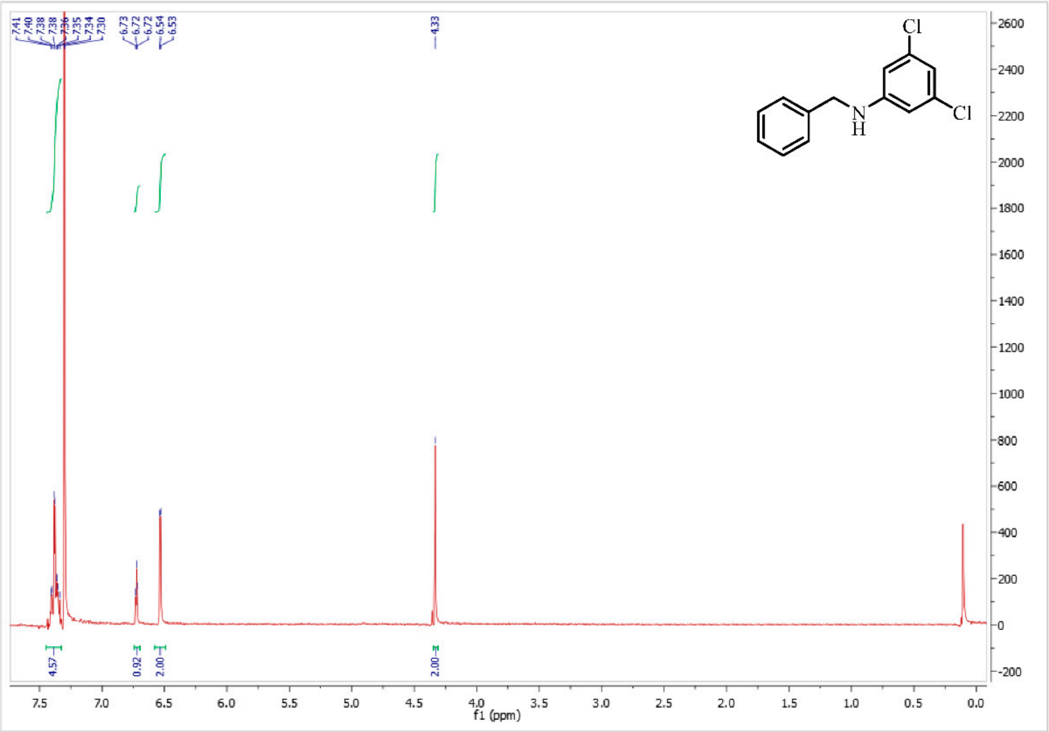

Figure S15. 3ai, ^{1}H-NMR.

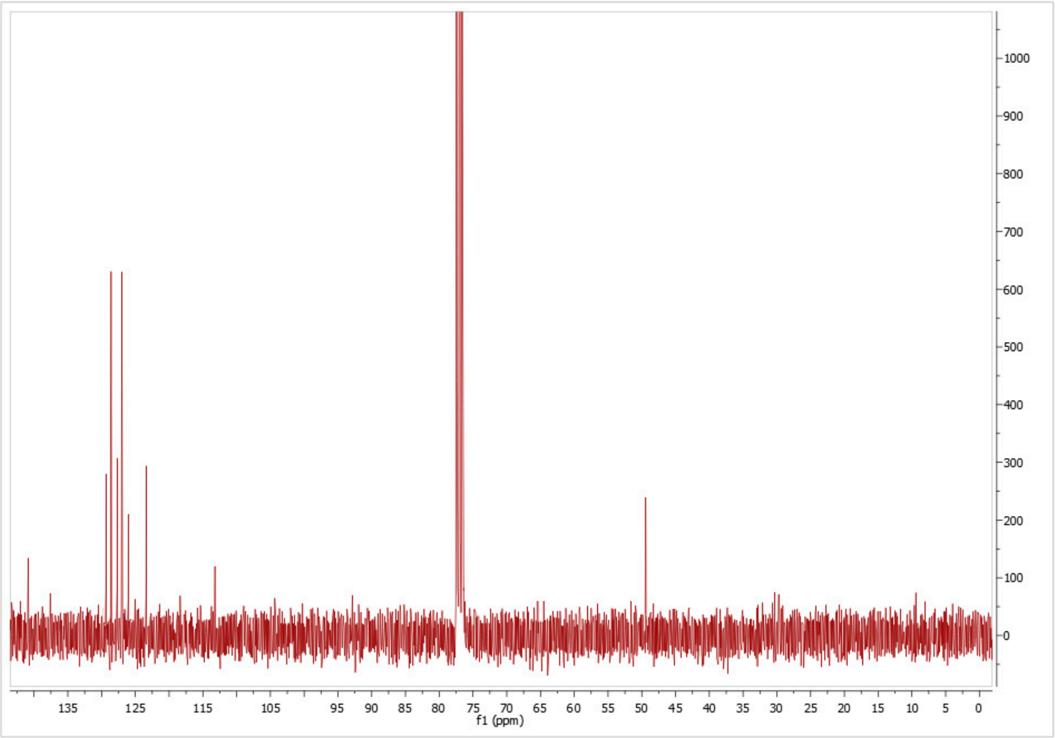

Figure S16. 3ai, ^{13}C-NMR.

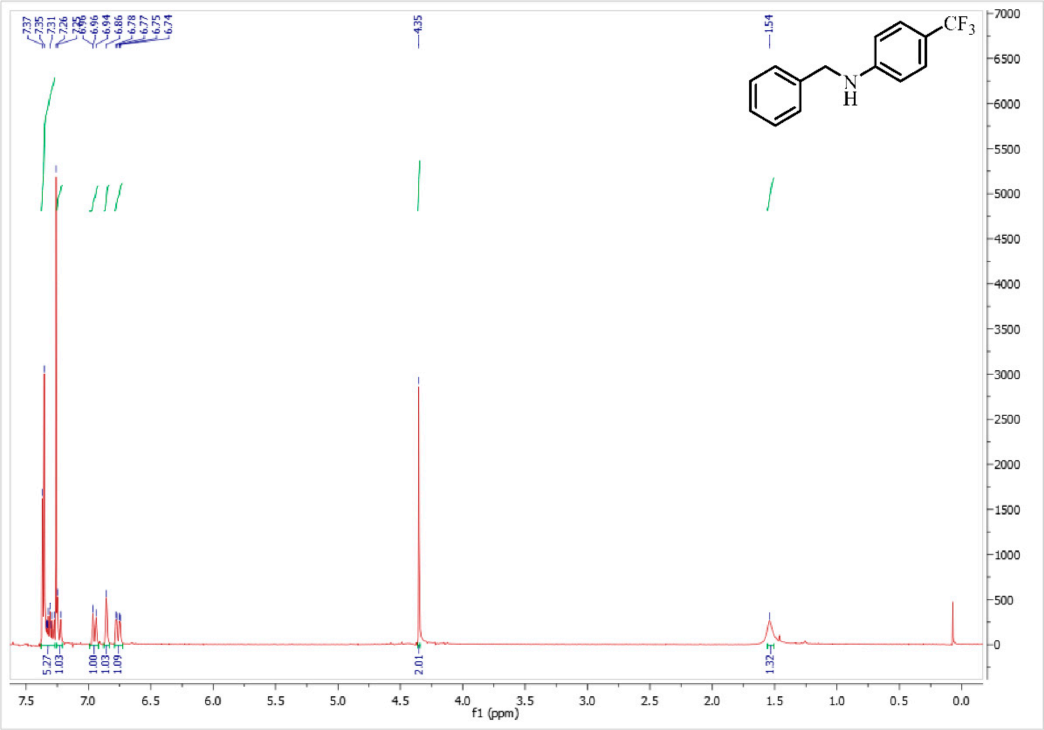

Figure S17. 3ak, ^{1}H-NMR.

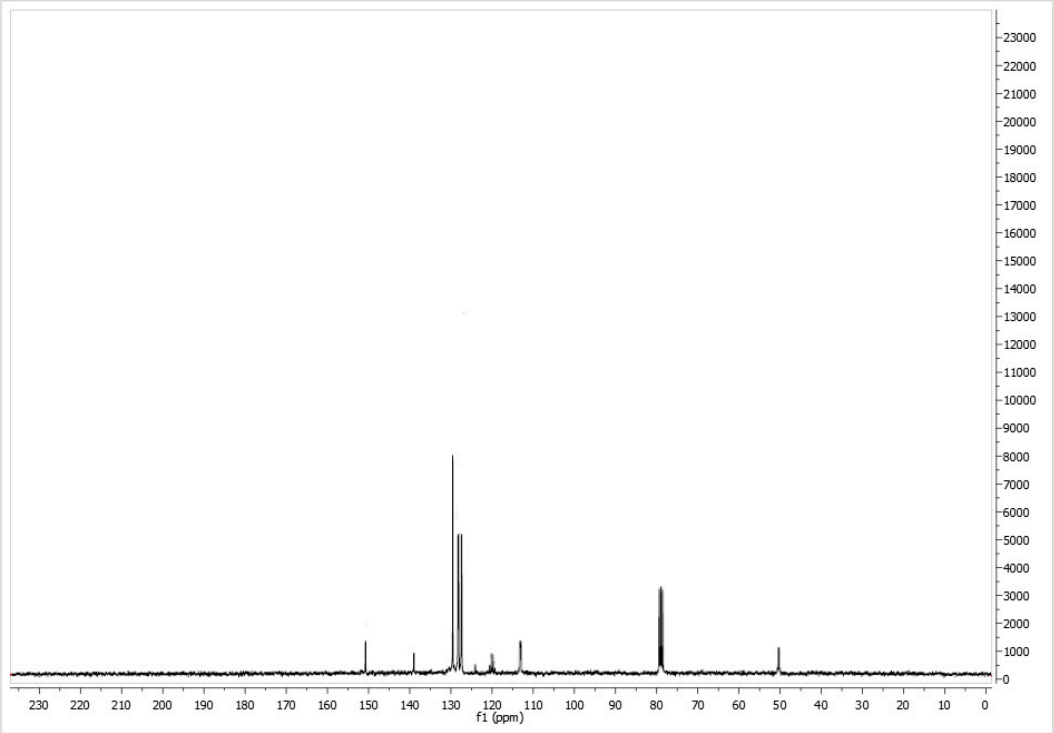

Figure S18. 3ak, ^{13}C-NMR.

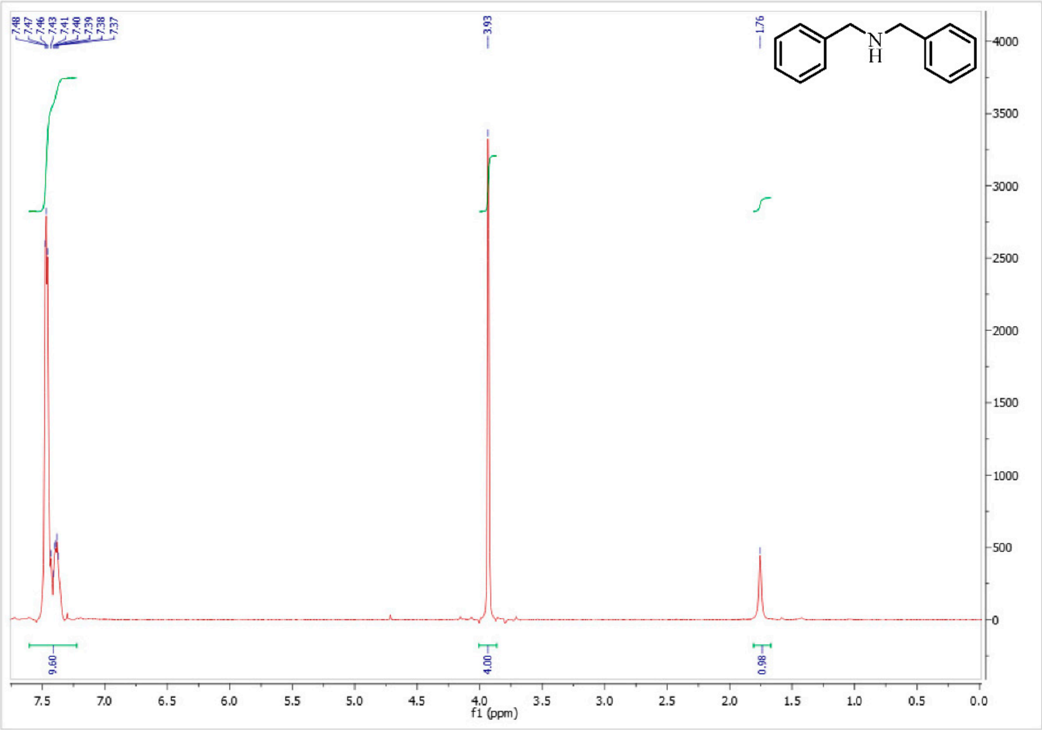

Figure S19. 3am, ^{1}H-NMR.

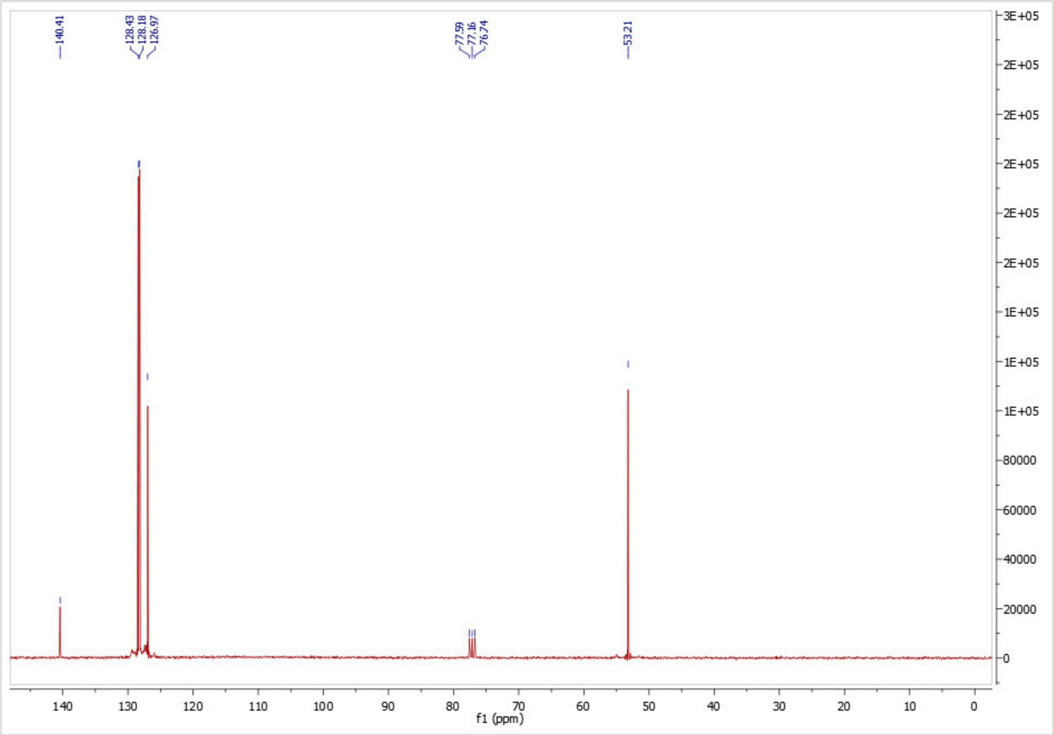

Figure S20. 3am, ^{13}C-NMR.

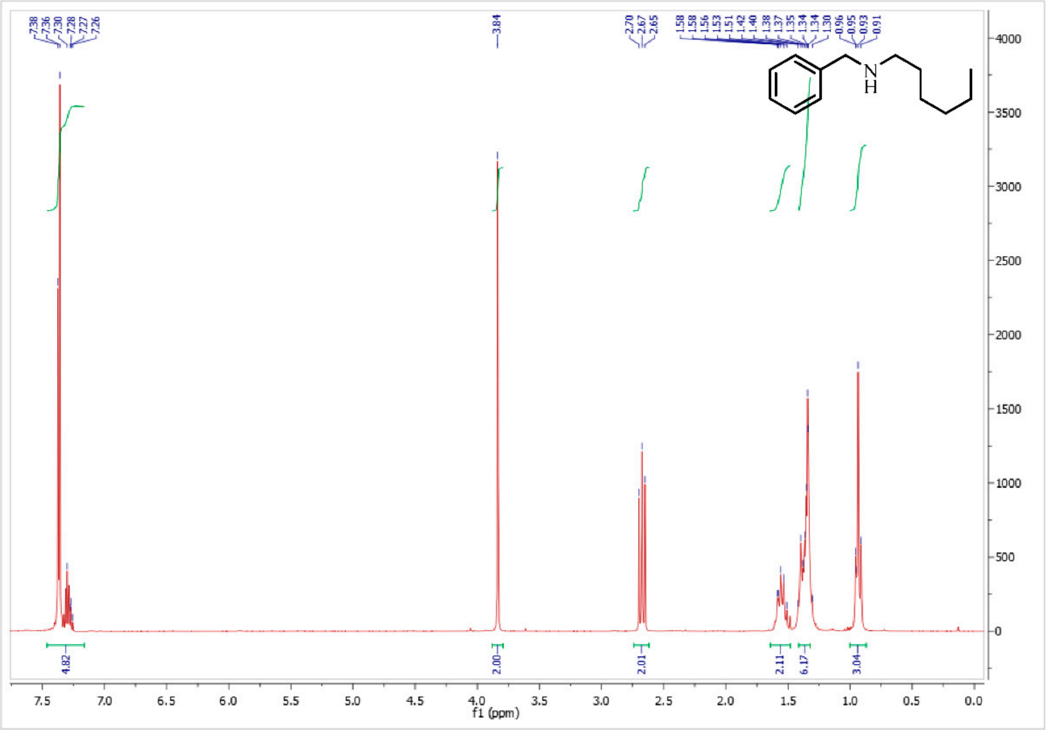

Figure S21. 3an, ^{1}H-NMR.

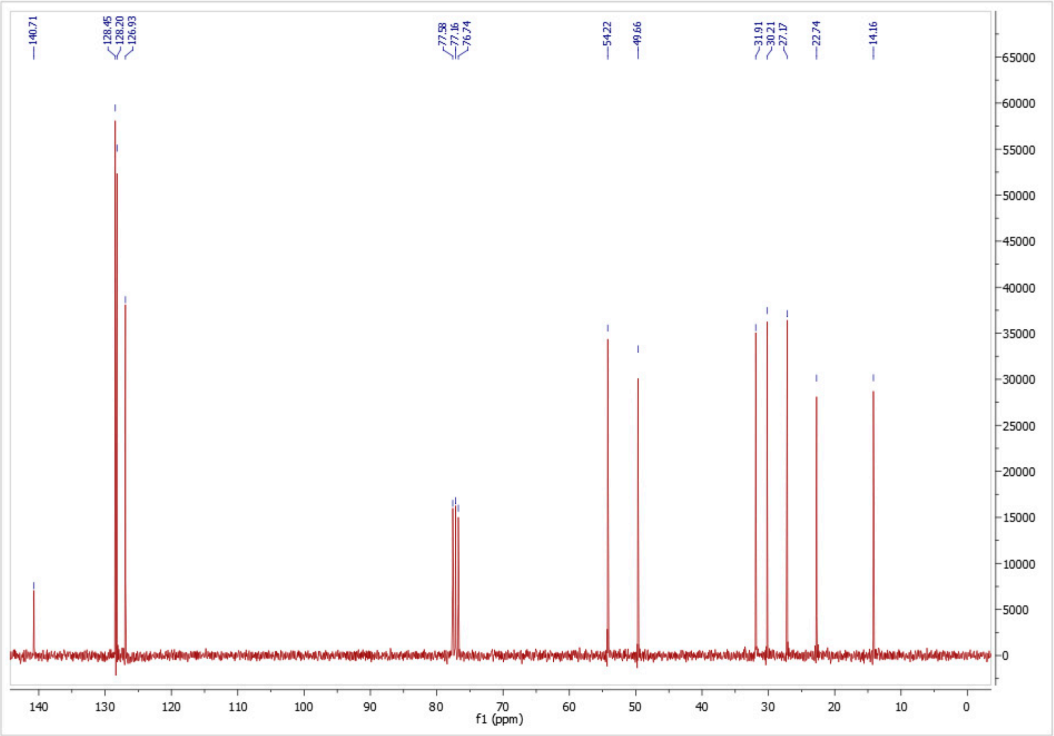

Figure S22. 3an, ^{13}C-NMR.

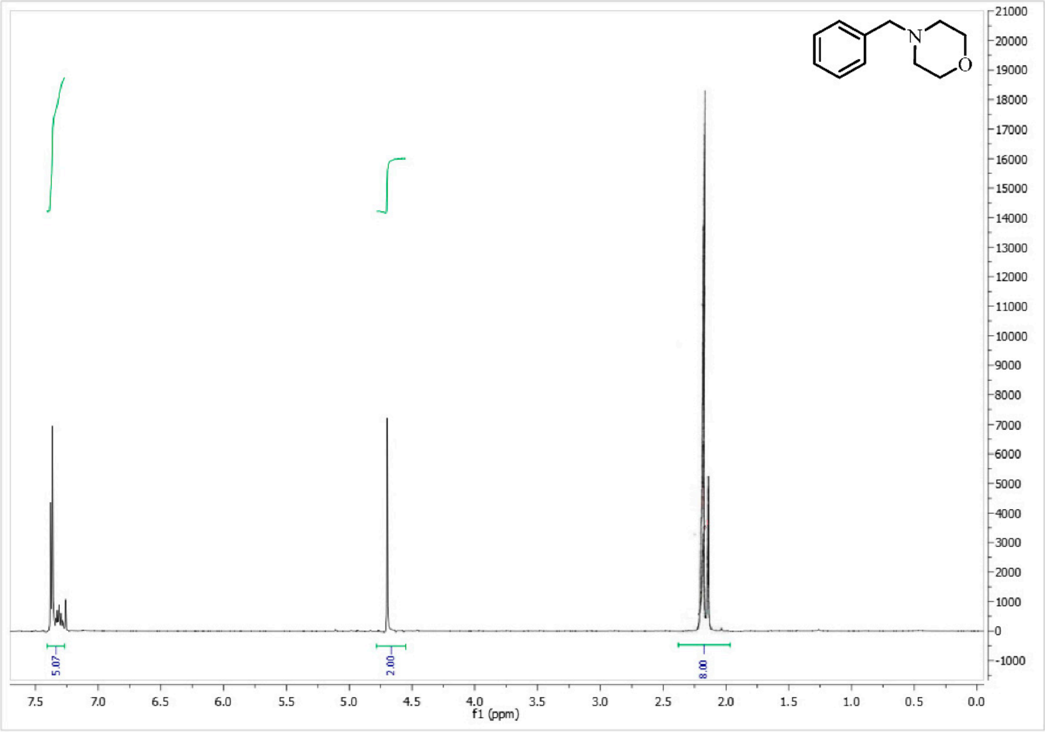

Figure S23. 3al, ^{1}H-NMR.

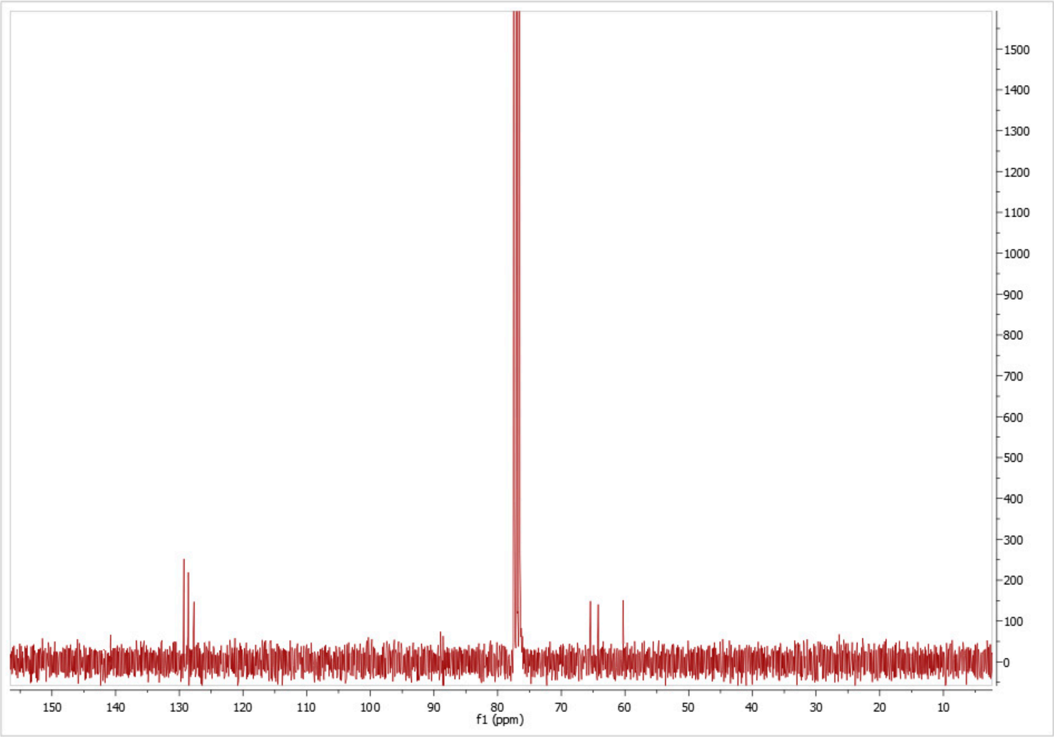

Figure S24. 3al, ^{13}C-NMR.

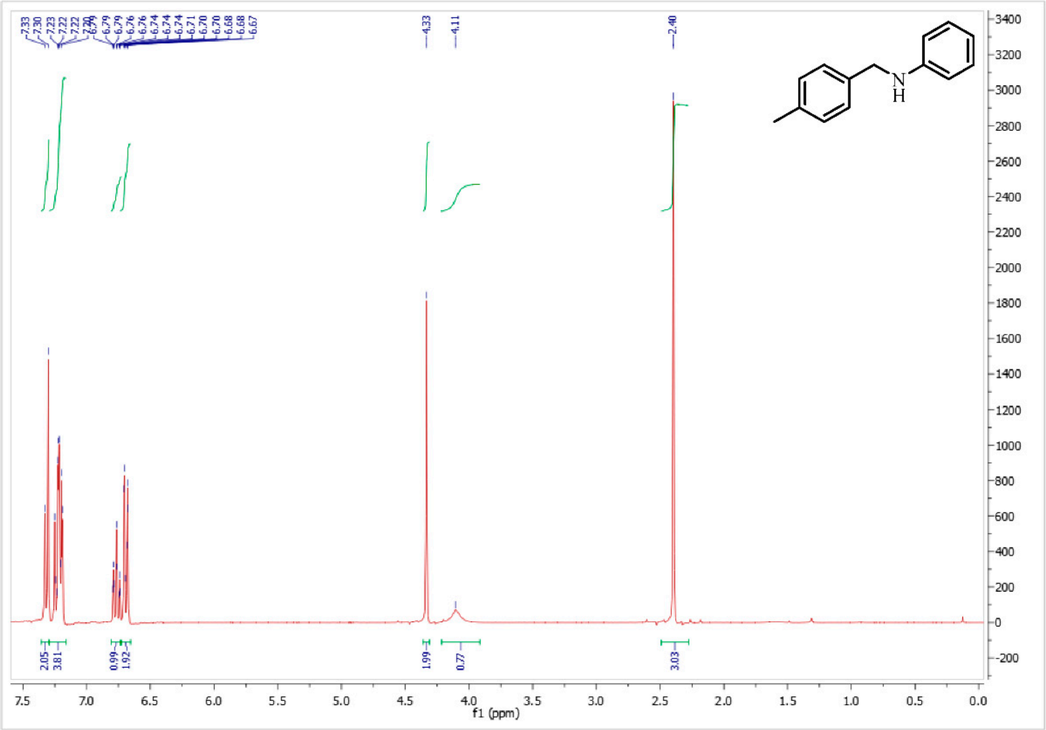

Figure S25. 3ba, ^{1}H-NMR.

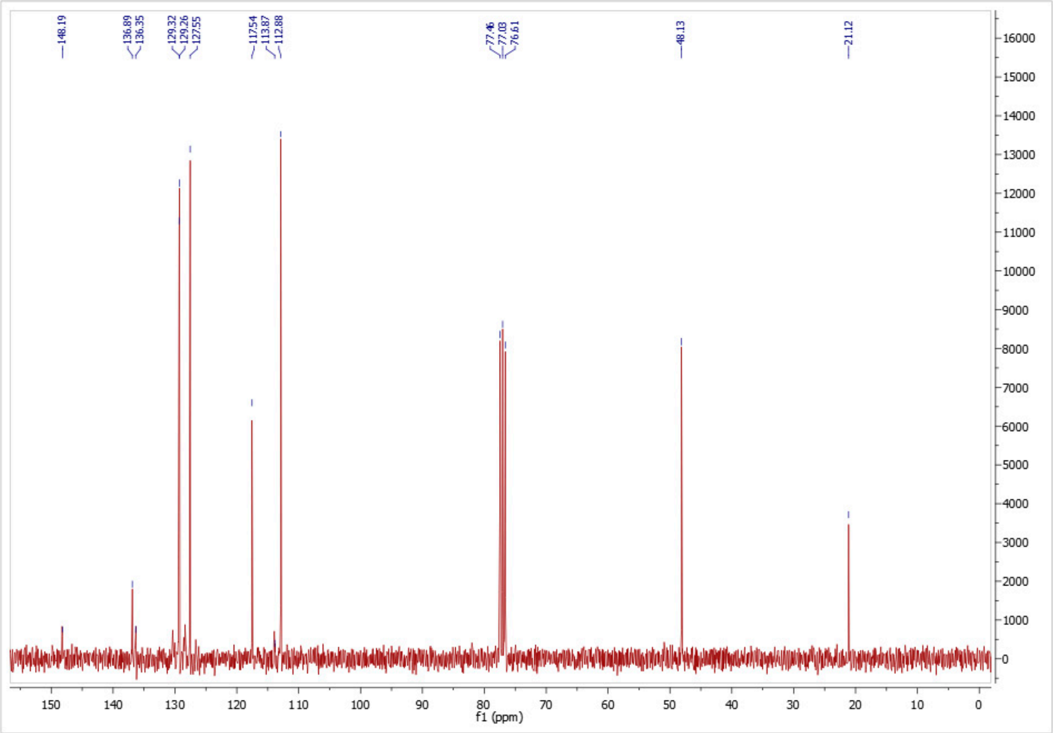

Figure S26. 3ba, ^{13}C-NMR.

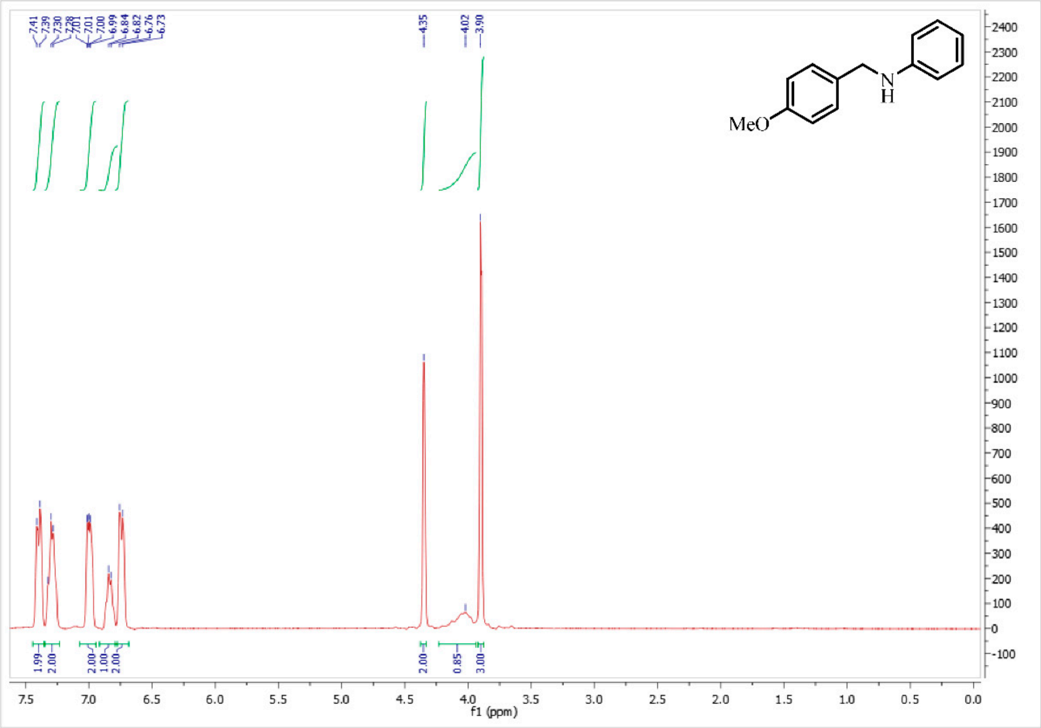

Figure S27. 3ca, ^{1}H-NMR.

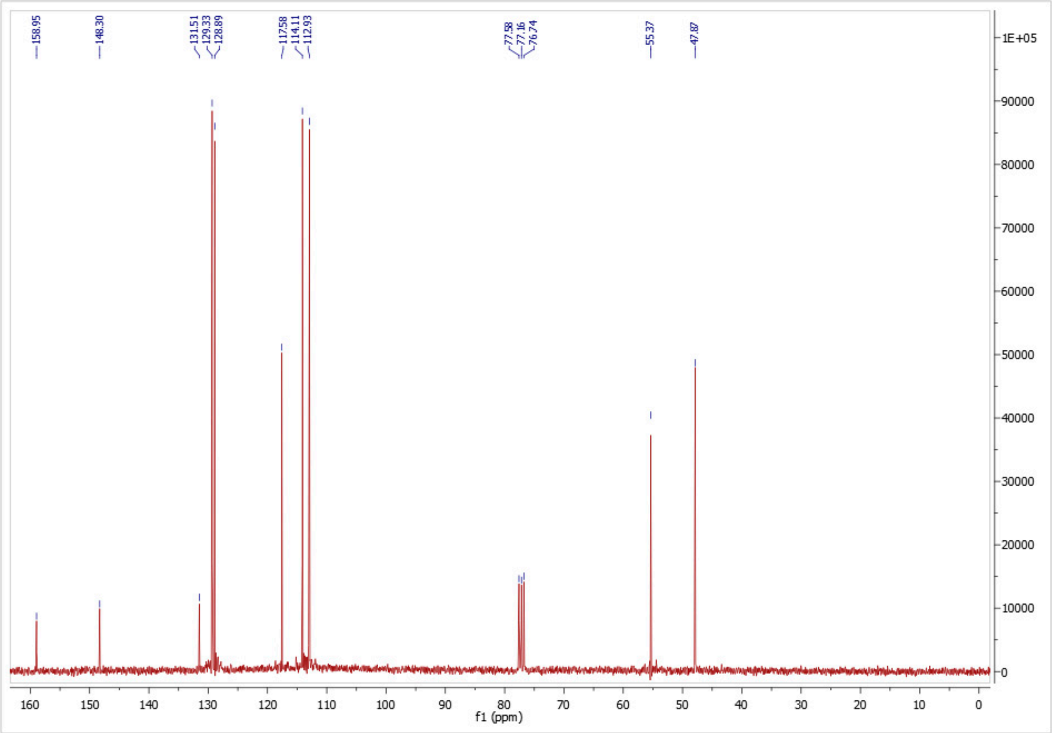

Figure S28. 3ca, ^{13}C-NMR.

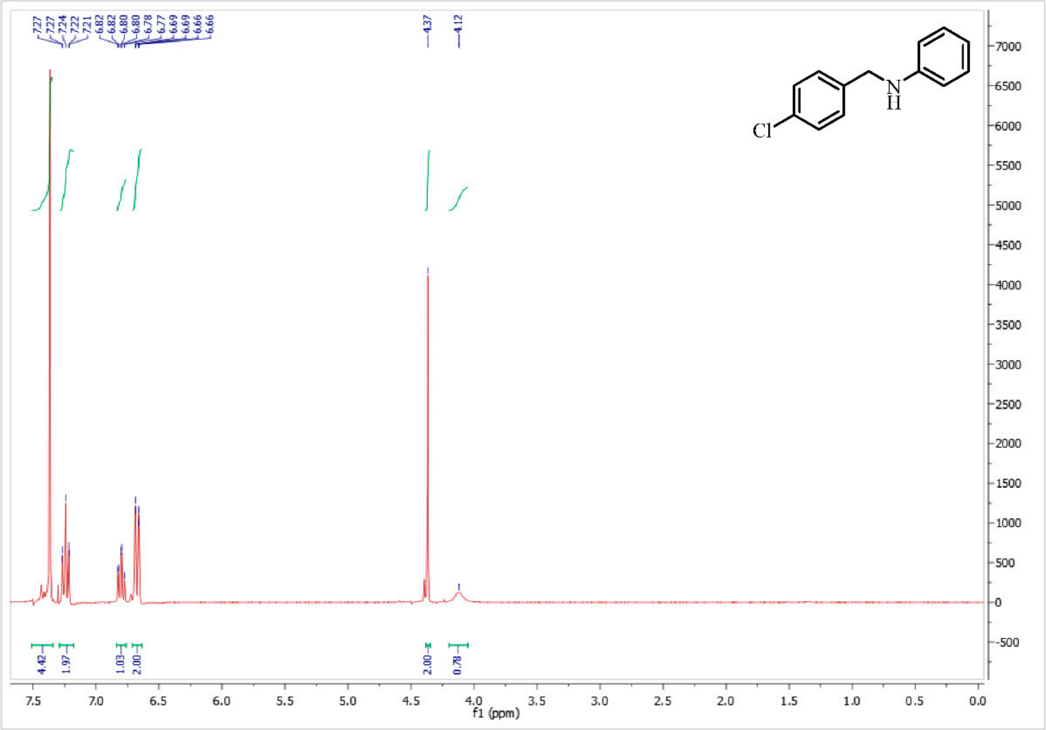

Figure S29. 3da, ^{1}H-NMR.

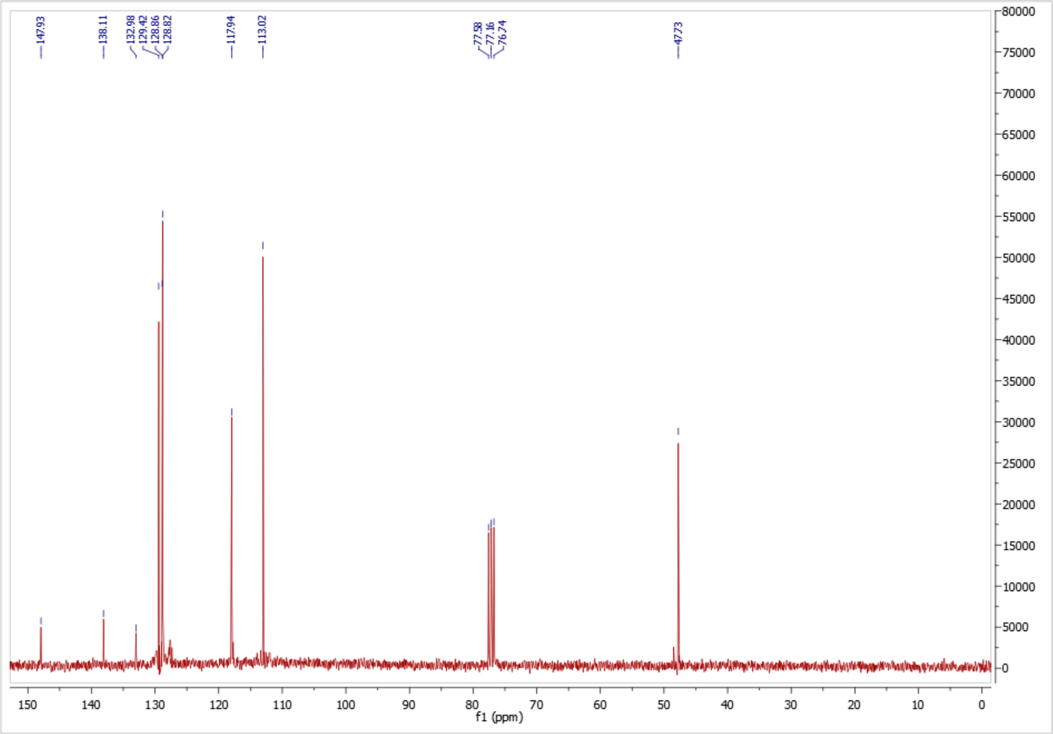

Figure S30. 3da, ^{13}C-NMR.

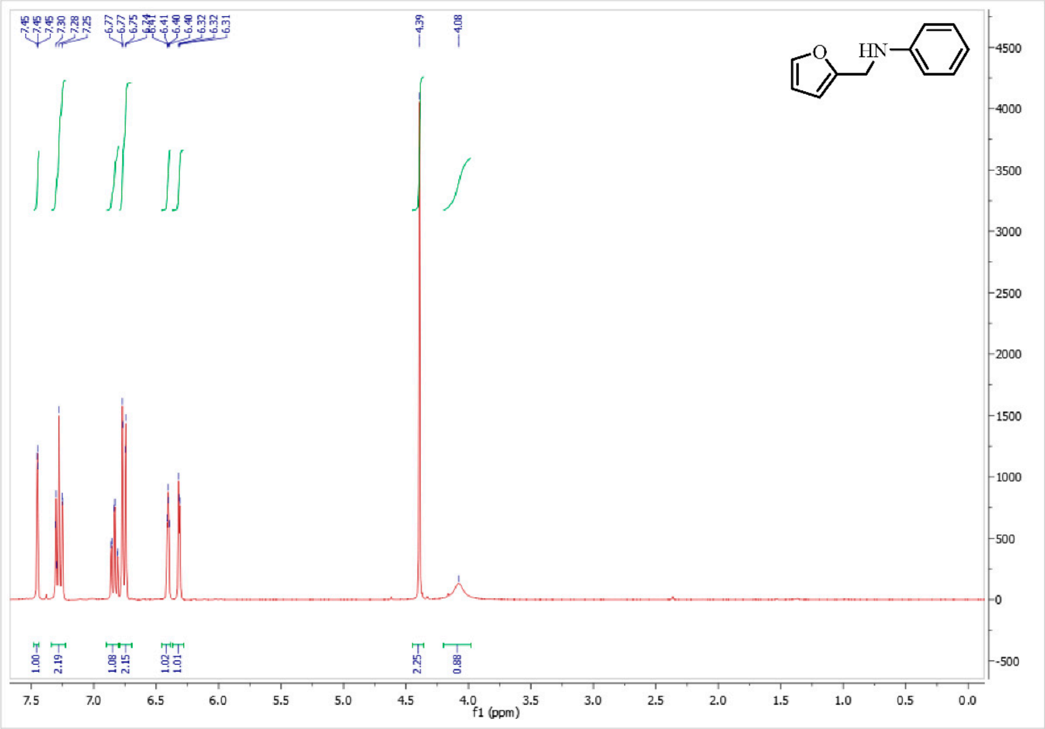

Figure S31. 3ea, ^{1}H-NMR.

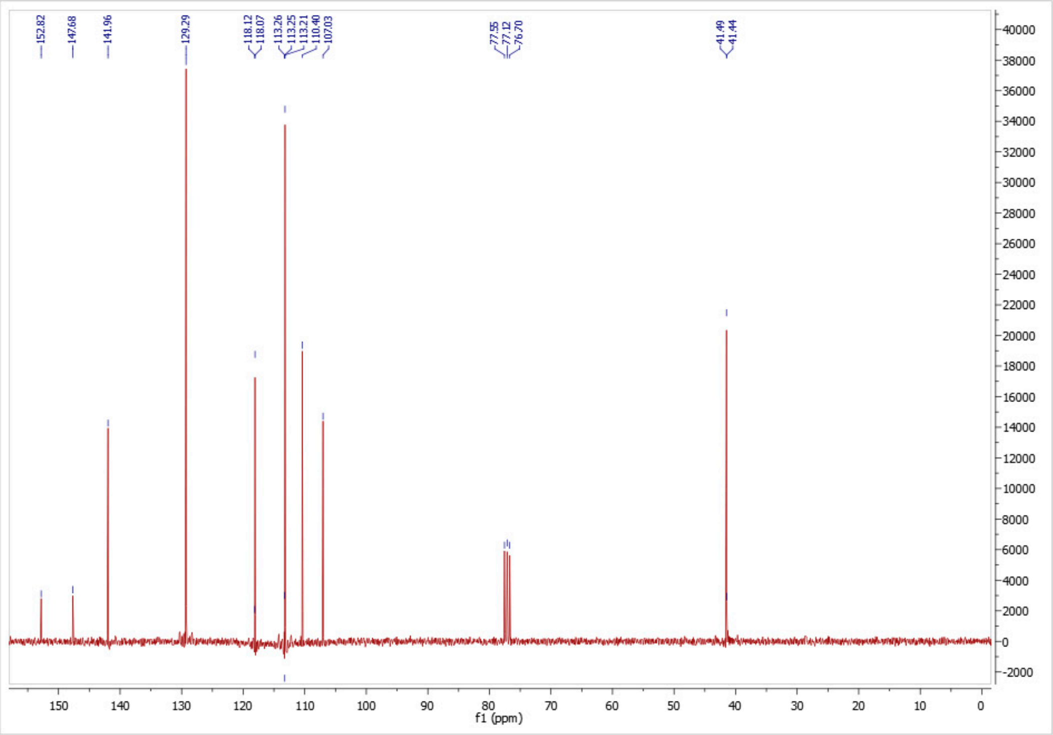

Figure S32. 3ea, ^{13}C-NMR.
